# Antiviral effects and tissue exposure of tetrandrine against SARS‐CoV‐2 infection and COVID‐19

**DOI:** 10.1002/mco2.206

**Published:** 2023-01-19

**Authors:** Jia Liu, Furun Wang, Xi Wang, Shiyong Fan, Yufeng Li, Mingyue Xu, Hengrui Hu, Ke Liu, Bohong Zheng, Lingchao Wang, Huanyu Zhang, Jiang Li, Wei Li, Wenpeng Zhang, Zhihong Hu, Ruiyuan Cao, Xiaomei Zhuang, Manli Wang, Wu Zhong

**Affiliations:** ^1^ State Key Laboratory of Virology Wuhan Institute of Virology Center for Biosafety Mega‐Science Chinese Academy of Sciences Wuhan China; ^2^ National Engineering Research Center for the Emergency Drug Beijing Institute of Pharmacology and Toxicology Beijing China; ^3^ Hubei Jiangxia Laboratory Wuhan China

**Keywords:** SARS‐CoV‐2, tetrandrine, antiviral efficacy, pharmacokinetics, tissue distribution, lung exposure

## Abstract

Tetrandrine (TET) has been used to treat silicosis in China for decades. The aim of this study was to facilitate rational repurposing of TET against SARS‐CoV‐2 infection. In this study, we confirmed that TET exhibited antiviral potency against SARS‐CoV‐2 in the African green monkey kidney (Vero E6), human hepatocarcinoma (Huh7), and human lung adenocarcinoma epithelial (Calu‐3) cell lines. TET functioned during the early‐entry stage of SARS‐CoV‐2 and impeded intracellular trafficking of the virus from early endosomes to endolysosomes. An in vivo study that used adenovirus (AdV) 5‐human angiotensin‐converting enzyme 2 (hACE2)‐transduced mice showed that although TET did not reduce pulmonary viral load, it significantly alleviated pathological damage in SARS‐CoV‐2‐infected murine lungs. The systemic preclinical pharmacokinetics were investigated based on in vivo and in vitro models, and the route‐dependent biodistribution of TET was explored. TET had a large volume of distribution, which contributed to its high tissue accumulation. Inhaled administration helped TET target the lung and reduced its exposure to other tissues, which mitigated its off‐target toxicity. Based on the available human pharmacokinetic data, it appeared feasible to achieve an unbound TET 90% maximal effective concentration (EC_90_) in human lungs. This study provides insights into the route‐dependent pulmonary biodistribution of TET associated with its efficacy.

## Graphical abstract

- Tissue concentrations were much higher than plasma concentration.
- Inhalation administration could enhance tetrandrine exposure in lungs.
- Unbound tetrandrine in human lungs may achieve unbound EC_90_ according to human pharmacokinetics in literature and *K*_p,lung_.

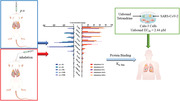

## 1 INTRODUCTION

The COVID‐19 pandemic is a public health concern worldwide. Although several small‐molecule drugs, including remdesivir, molnupiravir, nirmatrelvir, and ritonavir have been formally approved or granted emergency use authorization for the treatment of COVID‐19, SARS‐CoV‐2‐specific therapeutics that can prevent severe illness, hospitalization, and mortality are still in great demand.

Tetrandrine (TET) and cepharanthine are bisbenzylisoquinoline alkaloids extracted from herbal plants. Multiple studies have suggested that both compounds have anti‐inflammatory and immunoregulatory properties.^1^ Cepharanthine and TET have antiviral activity against human coronavirus OC43 (HCoV‐OC43) and SARS‐CoV‐2 in Vero cell screens.^2^ TET is a potent blocker of two‐pore iron channels (TPCs), which are located in the membranes of host endolysosomes (ELs) and are critical for viral entry. TET can inhibit infection by various viruses, including herpes simplex virus, dengue virus, Ebola virus, Middle East respiratory syndrome coronavirus (MERS‐CoV), and HCoV‐OC43.^3^, ^4^, ^5^ Additionally, TET has a broad spectrum of pharmacological activities, including anti‐cancer, anti‐inflammatory, immunosuppressant, anti‐fibrotic, and anti‐arthritic properties.^6^ Because the disease severity of COVID‐19 is linked to an excessive inflammatory response and pulmonary fibrosis,^7^ TET may potentially be used in the treatment of COVID‐19 due to its anti‐inflammatory and immunomodulatory effects. As one of the most promising antiviral candidates,^8^ TET has been reported to have a significant inhibitory effect against SARS‐CoV‐2 pseudotyped virus and authentic virus infection in Vero E6 cells.^5^, ^8^ However, the in vivo efficacy of TET in inhibiting SARS‐CoV‐2 replication and curing disease remains unclear.

In vivo exposure is the most important factor that affects drug efficacy. A 90%–99% reduction in viral replication is thought to be required for effective therapy of rapidly progressive acute viral infection.^9^ For example, the concentration required for a 90% reduction in SARS‐CoV‐2 virus replication (90% maximal effective concentration, EC_90_) is usually obtained using physiologically relevant cellular systems in the first phase.^9^ Although the significant role of EC_90_ in evaluating in vivo antiviral efficacy is well accepted, how to correctly compare the in vivo concentration with in vitro EC_90_ values warrants in‐depth investigation. Due to the inaccessibility of tissue concentration, plasma concentration is generally used as a surrogate for the concentration at the main site of action of the disease.^10^

In this study, the in vitro and in vivo anti‐SARS‐CoV‐2 activities of TET were evaluated, and the tissue exposure characteristics of TET in rats administered by different routes as single and multiple doses were determined. Particular attention was given to target exposure to TET in the lung tissue. The anti‐SARS‐CoV‐2 EC_90_ was correlated with unbound lung concentration. Based on the human pharmacokinetics (PK) of TET available in the literature, we highlighted the potential of TET for the treatment of COVID‐19.

## 2 RESULTS

### 2.1 Anti‐SARS‐CoV‐2 activity of tetrandrine in vitro

**FIGURE 1:**
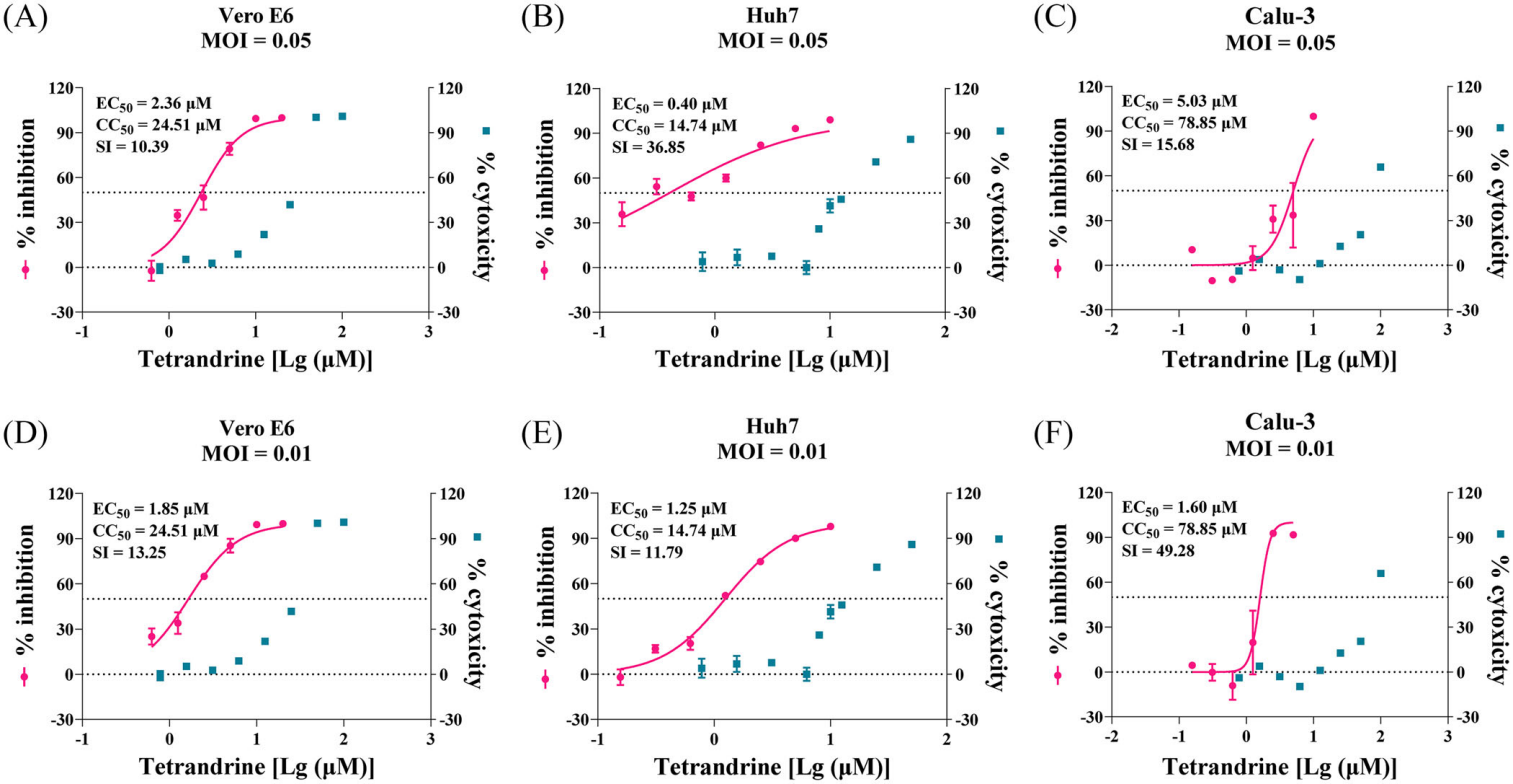
In vitro anti‐SARS‐CoV‐2 activity of tetrandrine in Vero E6 cells, human hepatocarcinoma cell line (Huh7), and human lung adenocarcinoma epithelial cells (Calu‐3). Cells were infected with SARS‐CoV‐2 at a multiplicity of infection (MOI) of either 0.05 (A–C) or 0.01 (D–F) and treated with different doses of tetrandrine for 24 h (Vero E6) or 48 h (Huh7 and Calu‐3). The viral yield in the supernatant was quantified by quantitative real‐time polymerase chain reaction (qRT‐PCR). CC_50_, 50% cytotoxic concentration; EC_50_, 50% maximal effective concentration; SI, selectivity index (SI = CC_50_/EC_50_)

The anti‐SARS‐CoV‐2 activity of TET was evaluated in three cell lines, including African green monkey kidney Vero E6 cells, human hepatocarcinoma cell line (Huh7), and human lung adenocarcinoma epithelial cells (Calu‐3). Firstly, the cytotoxicity of the drug in these cell lines was measured by cell‐counting kit‐8 (CCK8) assay. Then, the cells were infected with SARS‐CoV‐2 at a multiplicity of infection (MOI) of either 0.05 or 0.01 in the presence of TET or dimethyl sulfoxide (DMSO) as control. The dose–response curves were determined by detection of viral RNA levels by quantitative real‐time polymerase chain reaction (qRT‐PCR) in the supernatant of the infected cell at 24 or 48 h post‐infection (p.i.). As shown in the upper panel of Figure 1, TET efficiently inhibited SARS‐CoV‐2 infection (MOI = 0.05) in Vero E6 cells, with a 50% cytotoxic concentration (CC_50_) and 50% maximal effective concentration (EC_50_) of 24.51 and 2.36 µM, respectively, and a selectivity index (SI = CC_50_/EC_50_) of 10.39 (Figure 1A). TET also blocked virus infection in Huh7 and Calu‐3 cells with EC_50_ of 0.40 µM (CC_50_ = 14.74 µM, SI = 36.85) (Figure 1B) and 5.03 µM (CC_50_ = 78.85 µM, SI = 15.68) (Figure 1C). The calculated EC_90_ values were 7.65, 7.78, and 12.08 µM in Vero E6, Huh7, and Calu‐3 cells, respectively. The anti‐SARS‐CoV‐2 effect of TET at 0.01 MOI of SARS‐CoV‐2 infection was also measured (Figure 1D–F). The EC_50_ values of TET in Vero E6, Huh7, and Calu‐3 cells were 1.85, 1.25, and 1.60 µM, respectively. These results indicate that TET has direct antiviral activity against SARS‐CoV‐2 infection in vitro.

### 2.2 Tetrandrine functions through trapping virions within intracellular early endosomes

**FIGURE 2:**
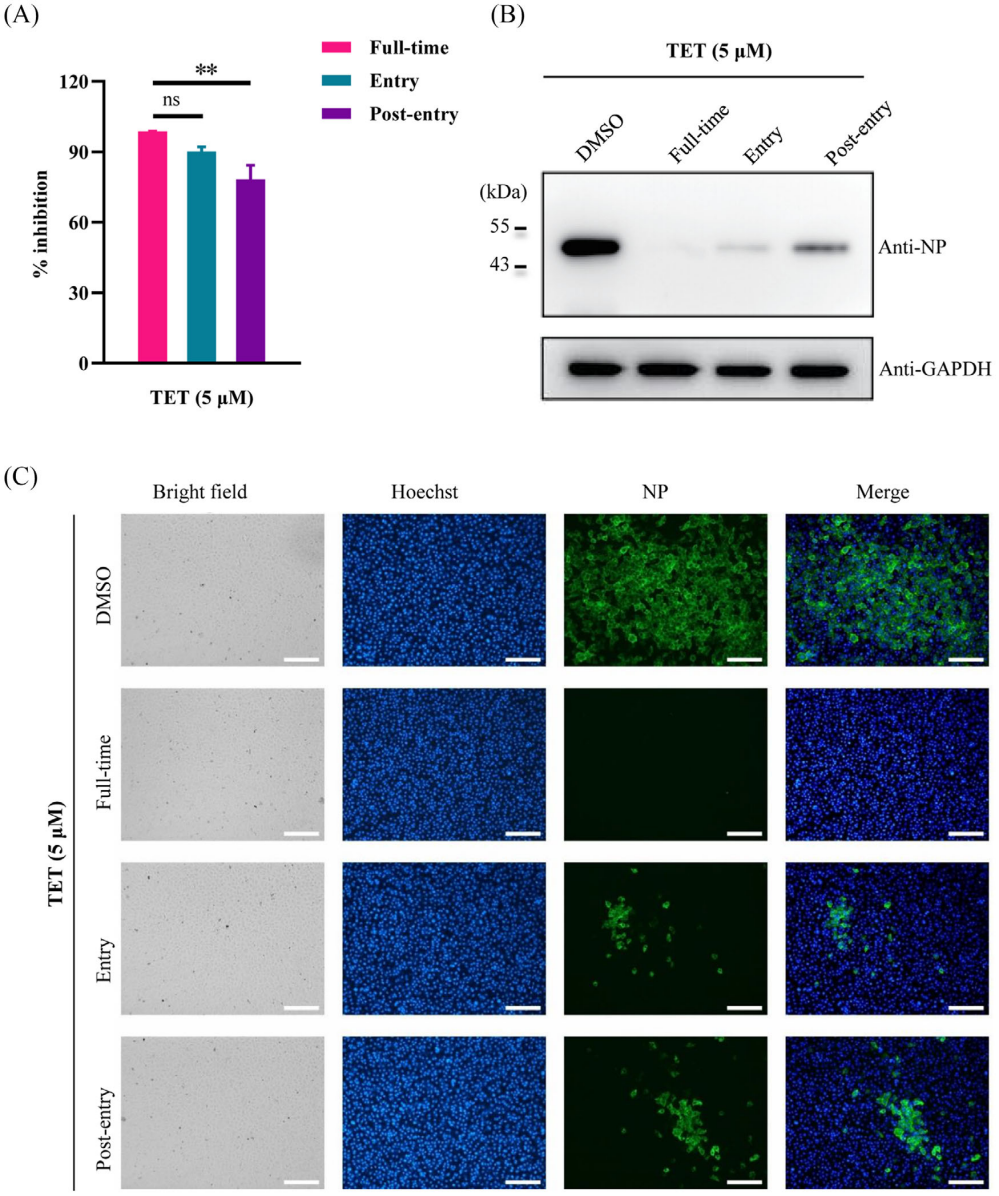
Time‐of‐addition assay of tetrandrine (TET). SARS‐CoV‐2‐infected Vero E6 cells were treated with TET at “full‐time”, “entry”, or “post‐entry” process. Virus yield in the infected cell supernatants was quantified by quantitative real‐time polymerase chain reaction (qRT‐PCR) (A) and nucleocapsid protein (NP) expression in infected cells was analyzed by western blotting (B) and immunofluorescence assay (C) at 16 h post‐infection (p.i.). Bar = 200 µm. ns indicates *p* > 0.05 and ^**^ *p* < 0.01

As shown in Figure 2, incubation with TET during virus entry and post‐entry processes potently inhibited virus production. TET was more effective for inhibiting production on virus entry (approximately 90% inhibition rate) than on the post‐entry process (approximately 70% inhibition rate) (Figure 2A). In addition, western blotting (Figure 2B) and immunofluorescence analysis (IFA) (Figure 2C) showed that the expression level of viral nucleocapsid protein (NP) was more markedly reduced at the entry stage than at the post‐entry stage.

**FIGURE 3:**
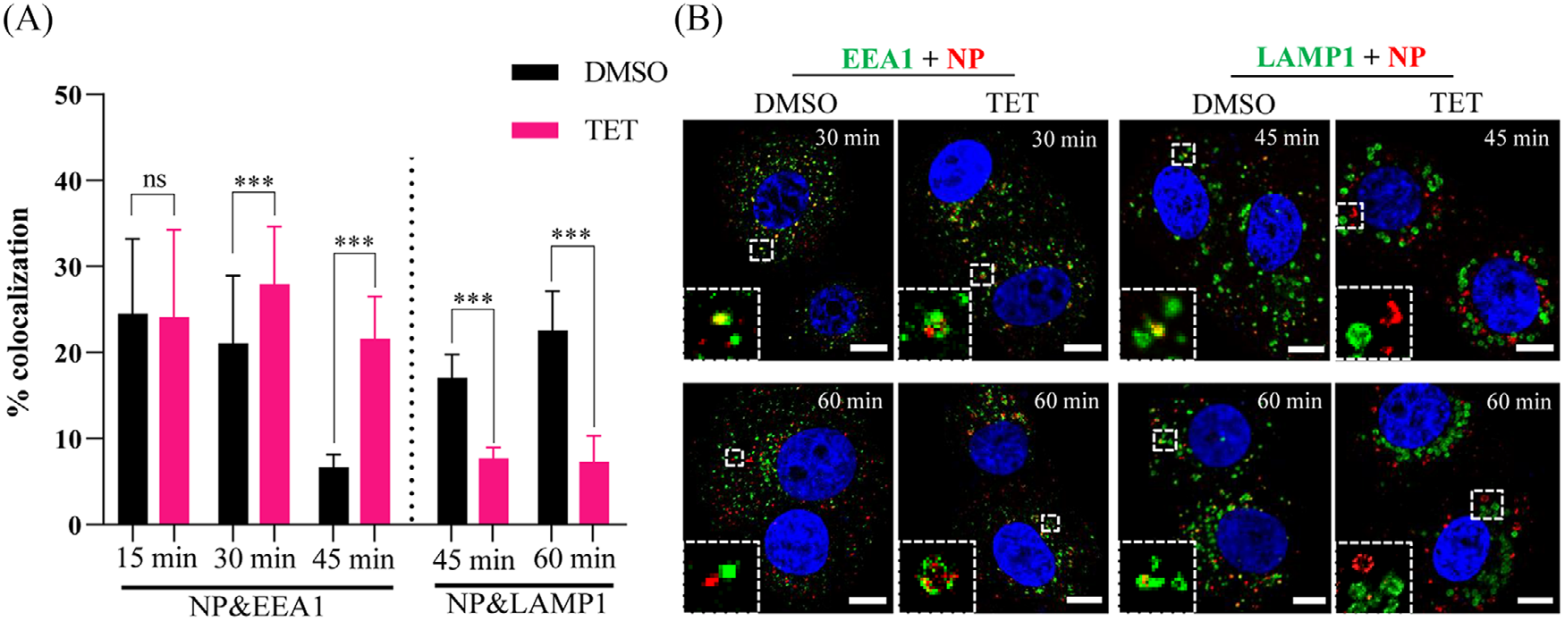
Mechanism of tetrandrine (TET) in inhibiting virus entry. Colocalization analysis of virions and endosomes was carried out at 15, 30, 45, or 60 min post‐infection through immunofluorescence assay using anti‐nucleocapsid protein (NP) antibody for virions (red) and anti‐early endosome antigen 1 (EEA1) antibody for early endosomes (EEs) (green) or anti‐lysosome‐associated membrane glycoprotein 1 (LAMP1) antibody for endolysosomes (ELs) (green). The nuclei (blue) were stained with 4',6‐diamidino‐2‐phenylindole. (A) The proportion of virions that colocalized with EEs or ELs in each group (*n* > 150 cells) was quantified. (B) Representative confocal microscopic images of viral particles (red), EEA1^+^ EEs (green), or LAMP1^+^ ELs (green) in each group are shown. The enlarged images of the small dashed boxes are inserted in the lower‐left corner of the same figure. Bar = 5 µm. ns indicates *p* > 0.05 and ^***^ *p* < 0.001

We further explored the mechanism of TET action as an inhibitor of endolysosomal ion channels in inhibiting viral entry by analyzing viral intracellular trafficking (Figure 3). Colocalization analysis (Figure 3A) of virions with early endosomes (EEs) or ELs showed that at 15 min p.i., the amounts of internalized virions located in early endosome antigen 1 (EEA1^+^) EEs were similar in the DMSO‐ and TET‐treated groups (24.5% and 24.1%, respectively). As the time of infection increased, the number of virions that colocalized with EEs decreased significantly in the DMSO group (21.1% and 6.7% at 30 and 45 min p.i., respectively), whereas increased internalized virions were observed in lysosome‐associated membrane glycoprotein (LAMP)^+^ ELs (17.5% and 22.6% at 45 and 60 min p.i., respectively), indicating that some virions had already been transported from EEs to ELs. In contrast, in the presence of TET, more virions were detected in the EEs (28.0% at 30 min and 21.6% at 45 min; *p* < 0.001), and less virions were located in the ELs (7.7% at 45 min and 7.3% at 60 min; *p* < 0.001), suggesting that TET trapped the virions in the EEs. In addition, TET treatment caused an enlargement and discontinuity of the membrane of the EE and EL vesicles (Figure 3B). Considered together, these results suggest that TET impedes intracellular trafficking of SARS‐CoV‐2 in endosomes.

### 2.3 Tetrandrine deceases the pathological lesions caused by SARS‐CoV‐2 infection in vivo

**FIGURE 4:**
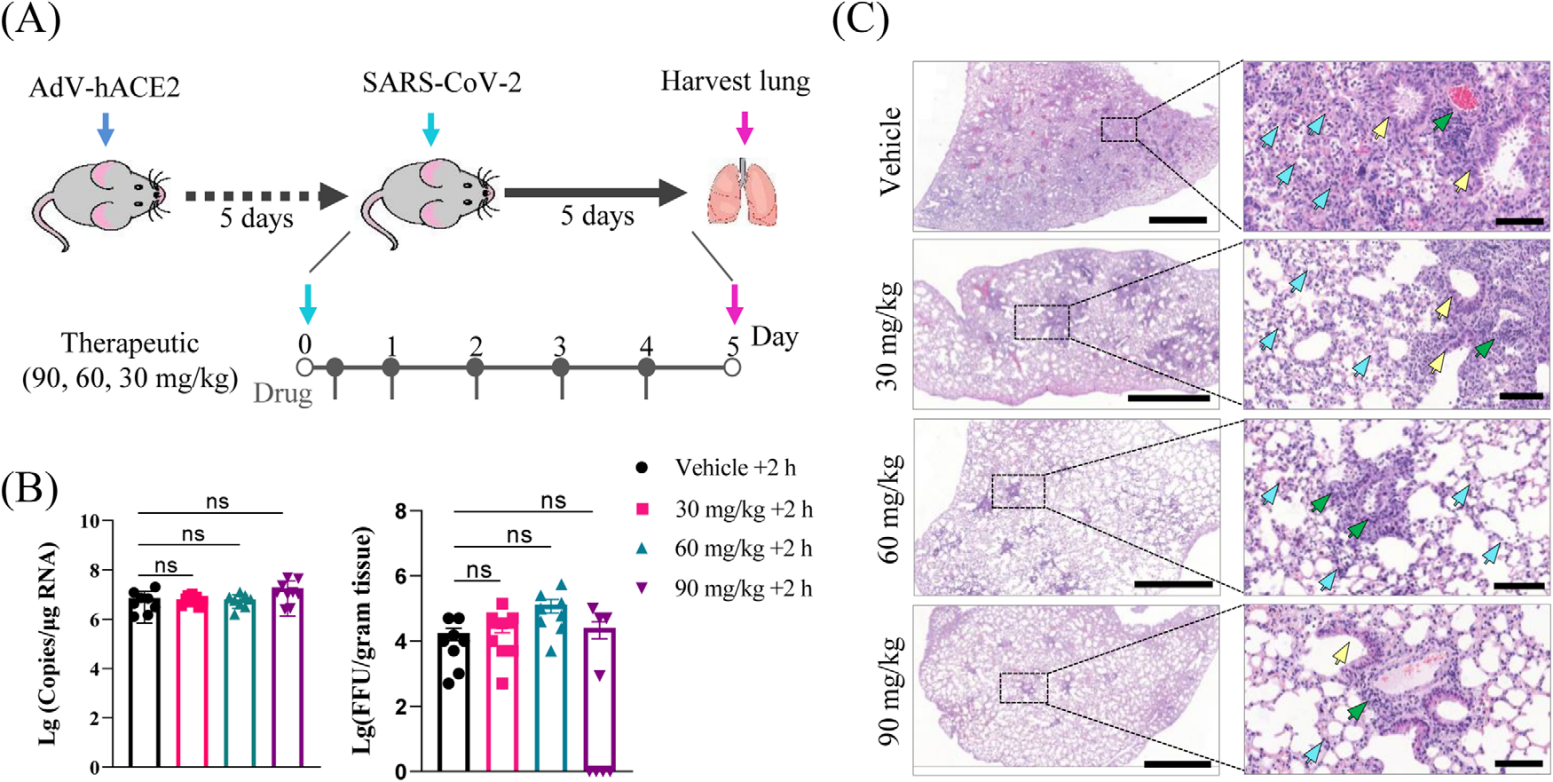
Therapeutic treatment with tetrandrine reduces SARS‐CoV‐2 disease in a mouse model. (A) Experimental design for evaluating the antiviral activity of tetrandrine (TET) in adenovirus (AdV) 5‐hACE2‐transduced type I interferon receptor knockout (IFNAR^−/−^) mice. Mice were injected intraperitoneally with 90, 60, or 30 mg/kg TET or vehicle (*n* ≥ 8 per group) at 2 h post‐infection (p.i.) (+2 h). (B) The viral RNA copies (left) and viral titer (right) in the lungs of infected mice. Data are mean ± standard error of mean (SEM). (C) Representative images of lung sections stained with hematoxylin and eosin. The images on the right (bars = 50 nm) are enlarged regions in the dashed boxes of the left images (bars = 1 µm). Blue, yellow, and green arrows show the pathological lesions in the alveoli, around the bronchi/bronchioles, and around blood vessels, respectively.

Adenovirus (AdV) 5‐hACE2‐transduced type I interferon receptor knockout (IFNAR^−/−^) mice were used to evaluate the therapeutic potential of TET against SARS‐CoV‐2 infection in vivo. This infection model has been widely used for evaluation of SARS‐CoV‐2 antivirals^11^, ^12^, ^13^, ^14^ (Figure 4A). Neither the viral RNA levels (Figure 4B, left) nor the infectious viral yields (Figure 4B, right) in the lungs decreased significantly after TET treatment at any of the three drug doses tested. However, histopathology analysis (hematoxylin and eosin staining) showed that TET alleviated pathological lesions in the lungs of SARS‐CoV‐2‐infected mice. As shown in Figure 4C, at 5 days p.i., the lungs of the control mice displayed severe diffuse alveolar damage (DAD), with marked thickening of alveolar septa, extensive exudation of protein in the alveoli, obvious epithelial hyperplasia in the bronchi and bronchioles, and extensive immune infiltration in the alveoli and around the bronchi and vessels. In contrast, in the mice treated with 90 or 60 mg/kg of TET, only slight lung damage was observed at 5 days p.i., as evidenced by the intact alveolar septa and cavities, and small numbers of immune cell infiltrate around the bronchi/bronchioles and blood vessels. In the low‐dose group (30 mg/kg), mild/moderate levels of DAD and interstitial inflammation were observed (Figure 4C).

### 2.4 Pharmacokinetic behavior of tetrandrine in rats

TET alleviated lung pathology without decreasing the viral load, suggesting that it would be useful to assess the PK properties of TET in vivo and explore the optimal strategy of drug administration for SARS‐CoV‐2 infection.

Liquid chromatography–mass spectrometry/mass spectrometry (LC‐MS/MS) methods of measuring TET in rat plasma and tissue homogenates were validated for selectivity, accuracy, precision, matrix effect, recovery, and stability. Calibration curves ranged from 2 to 2000 ng/ml in plasma and 5 to 2000 ng/ml in tissue homogenates. The intra‐day and inter‐day accuracy and precision met the standards for four different quality control concentrations. The relative standard deviations of the matrix effects were within 11%. The results showed that TET was stable in the matrix under various storage conditions (detailed bioanalysis methods are available in Supporting Information; the corresponding results are shown in Table S1–S3 and Figure S2).

**FIGURE 5:**
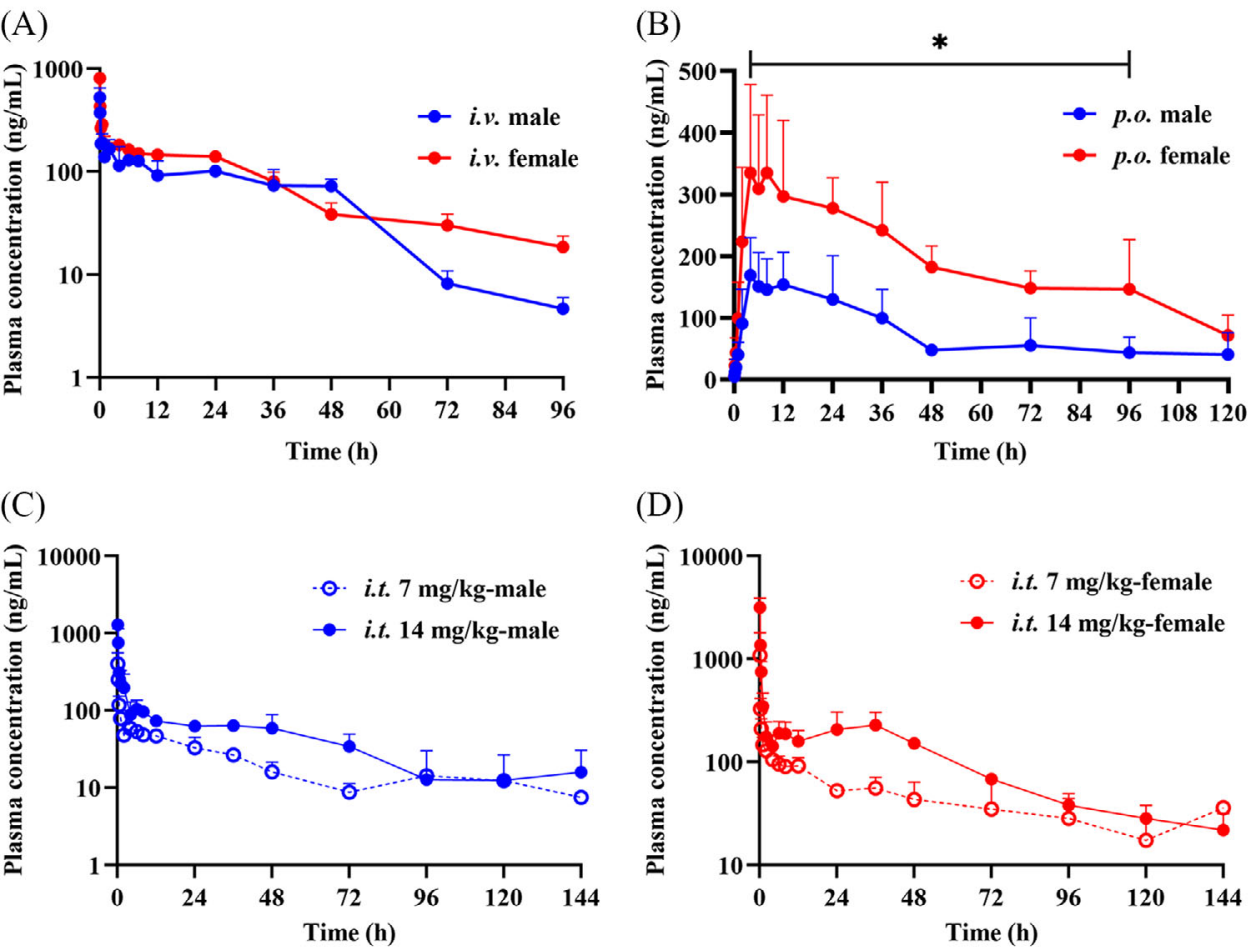
Mean plasma concentration versus time profiles of tetrandrine (TET) in rats via intravenous (i.v.), oral (per os, p.o.), or intratracheal (i.t.) administration. (A) This group of rats was administered with TET by i.v. injection at 5 mg/kg (*n* = 6, male:female = 1:1). (B) This group of rats was intragastrically administered 30 mg/kg TET suspended in 0.5% carboxymethyl cellulose sodium (CMC‐Na) solution (*n* = 6, male:female = 1:1). (C) Two groups of male rats were administered i.t. TET (simulating drug delivery by inhalation) at 7 and 14 mg/kg (*n* = 3). (D) Two groups of female rats were administered i.t. TET at 7 and 14 mg/kg (*n* = 3). All values are reported as mean ± standard deviation (SD). ^*^ *p* < 0.05 compared to concentrations in male rats by *t*‐test

The mean plasma concentration–time curves after single intravenous (i.v., 5 mg/kg), oral (per os [p.o.], 30 mg/kg), and intratracheal (i.t., 7 and 14 mg/kg) administration are shown in Figure 5. There was a significant difference in plasma concentrations between male and female rats, regardless of the dosing route. The plasma concentration in female rats was much higher than that in male rats. The major PK parameters of male rats are presented in Table S4. TET had low plasma clearance (CL, 13.6 ml/min/kg) and a large volume of distribution (*V*_d_, 24.8 L/kg) in male rats. Following oral administration, TET was absorbed slowly (*T*_max_ = 6.7 h), with moderate bioavailability (*F* = 33.6%) in rats. Compared to the fraction of absorbed (*F*_a_ × *F*_g_) (36.5%), hepatic metabolism played a minor role in the oral first‐pass effect on TET. TET was rapidly absorbed (*T*_max_ = 0.083–0.139 h) via i.t. administration compared to p.o. administration. The plasma concentration declined quickly after i.v. and i.t. administration in the distribution phase, but decreased smoothly in the elimination phase, indicating that TET is distributed rapidly to tissues and released slowly from tissues into the blood.

### 2.5 Route‐dependent tissue distribution of TET in rats

**FIGURE 6:**
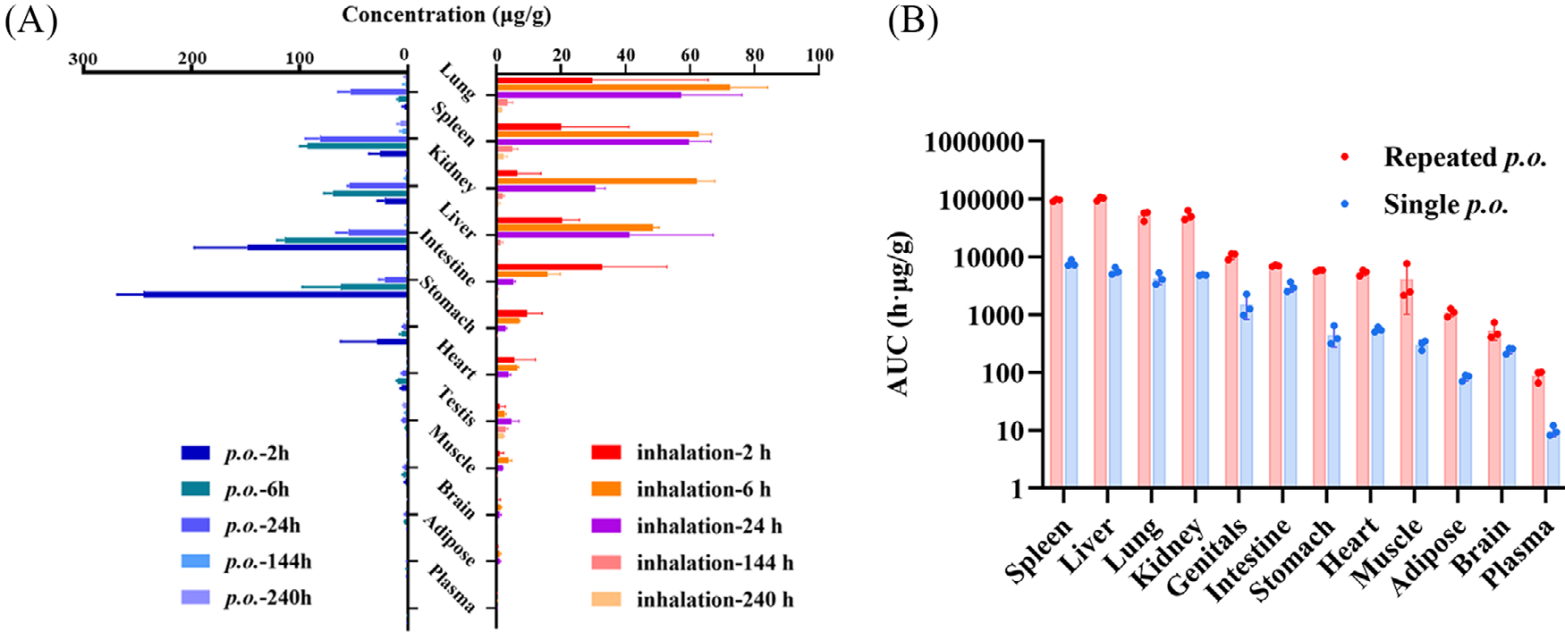
Comparative biodistributions of tetrandrine (TET) in major tissues in male rats via different dosing administration forms. (A) Five groups of rats received a single oral administration of TET (30 mg/kg) suspended in 0.5% carboxymethyl cellulose sodium (CMC‐Na) solution (30 mg/kg, left); five groups of rats received a single intratracheal (i.t.) dose of TET (7 mg/kg, right). (B) Mean area under the curves (AUCs) of TET in different tissues after single or repeated oral administration of TET suspended in 0.5% CMC‐Na solution (30 mg/kg) in rats. All AUCs of individual tissues have significant differences between repeated per os (p.o.) and single p.o. (*p* < 0.05). All values are shown as mean ± standard deviation (SD) (*n* = 3)

The mean exposures of TET in various tissues after oral (single and repeated at 30 mg/kg) and i.t. (7 mg/kg) administration are shown in Figure 6. Taking tissue protein binding rates into consideration (Table S5), it was found that the biodistribution of TET in tissues showed three characteristics (Tables 1 and S6–S8). First, the exposure in tissues was much higher than that in plasma, with unbound tissue partition ratio (*K*_p_) values >1, which is consistent with the large volume of distribution (*V*_d_ = 24 L/kg) obtained after i.v. administration. Second, the amount of exposure in various tissues could be sorted into three tiers: high level with *K*_p,uu_ > 20 (including the lung, spleen, liver, and kidney), moderate level with 2 < *K*_p,uu_ < 10 (including the stomach, muscle, heart, and testis), and low level with *K*_p,uu_ < 2 (including the brain, intestine, and adipose tissue). Third, the rank orders of TET biodistribution were similar in the three dosing patterns, with higher accumulation in the lung in each case. However, inhalation of TET led to the much higher exposure in the lung and relatively lower liver exposure compared to oral administration. Although high lung exposure to TET is the desired outcome, TET also causes hepatic toxicity. However, our results showed that inhalation could improve lung exposure to TET at lower dose levels, which might achieve a local effect without side effects.

**TABLE 1:** Pharmacokinetic parameters of tetrandrine in five major tissues and plasma after oral (per os, p.o.) and intratracheal (i.t.) administration in male rats

Tissue	Dosage and route of administration	*T*_max_ (h)	*C*_max_ (µg/ml)	AUC_(0–_ *_t_*_)_ (h µg/ml)	AUC_(0–∞)_ (h µg/ml)	*K*_p_	*K*_p_ ratio
Spleen	Single p.o.	6.00 ± 0	92.6 ± 7.23	7561 ± 906	7817 ± 987	802 ± 78.7	1
	Repeated p.o.	6.00 ± 0	445 ± 17.7	94,730 ± 5418	96,692 ± 4823	1136 ± 319	1.42
	Single i.t.	12.0 ± 10.4	64.5 ± 4.77	5517 ± 584	5676 ± 604	712 ± 210	0.888
Liver	Single p.o.	3.33 ± 2.31	153 ± 42.1	5695 ± 840	5727 ± 838	603 ± 186	1
	Repeated p.o	3.33 ± 2.31	938 ± 18.9	102,266 ± 7472	102,729 ± 7482	1213 ± 384	2.01
	Single i.t.	12.0 ± 10.4	54.6 ± 11.3	3613 ± 1747	3638 ± 1734	444 ± 232	0.736
Lung	Single p.o.	24.0 ± 0	53.5 ± 10.9	4182 ± 967	4273 ± 992	458 ± 187	1
	Repeated p.o	6.00 ± 0	337 ± 89.4	52,196 ± 10,257	52,910 ± 10,023	628 ± 235	1.37
	Single i.t.	4.67 ± 2.31	76.2 ± 5.51	5286 ± 1379	5381 ± 1387	662 ± 200	1.44
Kidney	Single p.o.	6.00 ± 0	69.8 ± 7.77	4815 ± 71.8	4870 ± 82.4	508 ± 101	1
	Repeated p.o	4.67 ± 2.31	278 ± 23.2	51,827 ± 10,474	53,212 ± 10,125	643 ± 295	1.27
	Single i.t.	4.67 ± 2.31	70.8 ± 15.5	3150 ± 209	3204 ± 224	404 ± 127	0.795
Intestine	Single p.o.	2.00 ± 0	244 ± 24.0	3029 ± 596	3049 ± 592	322 ± 113	1
	Repeated p.o	2.00 ± 0	178.5 ± 21.1	7005 ± 227	7055 ± 241	82.3 ± 19.1	0.256
	Single i.t.	3.33 ± 2.31	35.2 ± 16.8	693 ± 18.6	704 ± 11.3	90.4 ± 32.7	0.281
Plasma	Single p.o.	10.7 ± 11.7	0.180 ± 0.040	9.58 ± 2.10	9.85 ± 2.02	1	–
	Repeated p.o	6.00 ± 0	0.710 ± 0.280	87.7 ± 19.7	88.8 ± 20.2	1	–
	Single i.t.	6.67 ± 4.62	0.169 ± 0.611	8.99 ± 2.60	12.6 ± 6.15	1	–

Abbreviations: AUC, area under the curve; *C*_max_, maximum concentration; *K*_p_, tissue partition ratio.

### 2.6 In vitro metabolic stability and CYP reaction phenotyping

The metabolic stability of TET (1 µM) was measured in rat liver microsomes (RLM) and human liver microsomes (HLM) fortified with nicotinamide adenine dinucleotide phosphate (NADPH) using the substrate depletion method (Table S9). Intrinsic clearances (CL_int_) of TET in RLM (CL_int_ = 61.5 ml/min/mg protein) and HLM (CL_int_ = 46.7 ml/min/mg protein) were obtained. Hepatic blood clearance (CL_h,blood_) was calculated from CL_int_ using physiological constants from rats and humans, unbound fractions, and blood/plasma partitioning (*R*_b/p_, 3.08, measured in rat blood and used as a surrogate for humans). The extrapolated CL_h_ values for rats (CL_h_ = 1.8 ml/min/kg) and humans (CL_h_ = 0.628 ml/min/kg) were very low.

The metabolic turnover of TET was also measured using a panel of recombinant P450s. As shown in Figure S3A, incubation of TET with human CYP3A4 and CYP3A5 led to depletion of the parent drug. According to the total normalized rate (TNR) approach, the metabolic contributions of CYP3A4 and CYP3A5 were 88.3% and 11.7%, respectively.

### 2.7 Correlation between unbound tetrandrine concentration and 90% maximal effective concentration

**FIGURE 7:**
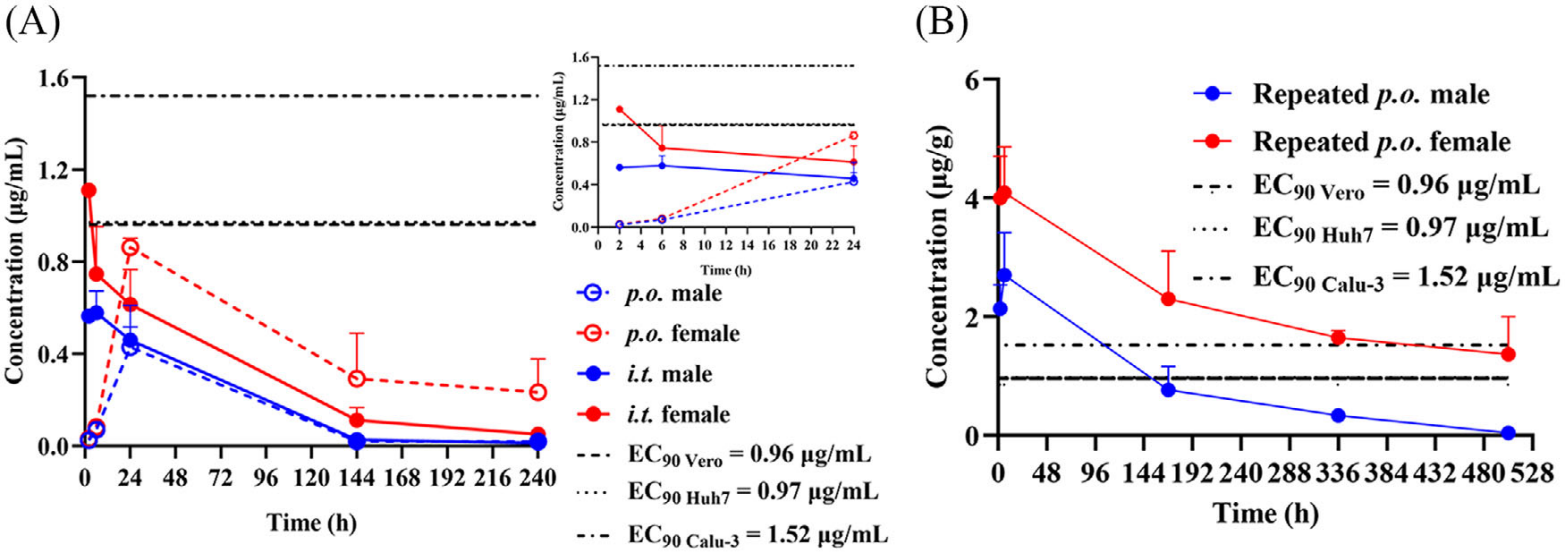
Effective pulmonary exposures of tetrandrine (TET). (A) Unbound TET pulmonary concentration–time profiles in rats after single oral (per os, p.o.) (30 mg/kg) and intratracheal (i.t.) (7 mg/kg) dosing of TET compared with three unbound antiviral 90% reduction in virus replication (EC_90_) values in different cells. The smaller panel is the unbound TET pulmonary concentration–time profiles in the first 24 h. (B) Unbound TET pulmonary concentration–time profiles in rats after multiple oral doses of TET (30 mg/kg) compared with unbound antiviral EC_90_ values in different cells (*n* = 3 for male or female rats). All values are shown as mean ± standard deviation (SD)

Given that only unbound drugs could exert a therapeutic effect by crossing the membrane barrier, the unbound EC_90_ obtained in Vero E6, Huh7, and Calu‐3 cells, and TET pulmonary concentrations were measured to estimate whether unbound TET in the lungs is effective against SARS‐CoV‐2. As the free fraction of TET was 0.202 in the culture medium (Table S5), the exact unbound EC_90_ values were 1.55 µM (0.96 µg/ml), 1.57 µM (0.97 µg/ml), and 2.44 µM (1.52 µg/ml) in Vero E6, Huh7, and Calu‐3 cells, respectively. The unbound EC_90_ and unbound TET concentrations in the lungs after p.o. and i.t. administration are compared in Figure 7. The unbound TET in the lungs after a single oral dose of 30 mg/kg was below the unbound EC_90_ threshold (Figure 7A). However, administering repeated oral doses of 30 mg/kg led to unbound TET levels in the lungs, considerably above the EC_90_, which was maintained for at least 96 h (Figure 7B). Compared with oral administration, the unbound TET concentration in the lungs was close to the EC_90_, and the TET level in other tissues was lower after i.t. administration at 7 mg/kg than after oral dosing. Therefore, it may be possible to achieve the efficacious EC_90_ in the lungs after i.t. administration, if the dosage, frequency, and interval are optimized.

## 3 DISCUSSION

To our knowledge, this is the first study to show that TET has anti‐SARS‐CoV‐2 potency in vitro and significantly alleviates murine lung injuries induced by SARS‐CoV‐2 infection. Although TET has been widely used to treat silicosis in China for nearly 50 years,^15^ there are few studies available on its systematic PK. The unavailability of data makes it difficult to bridge in vivo PK data with in vitro efficacy results according to the standard drug repositioning approach. In this study, plasma PK and tissue distribution results from different routes of administration in rats and data on in vitro protein binding fraction, metabolic stability, and therapeutic efficacy were integrated to measure the exposure–response relationships and identify the optimal administration route of TET to achieve preferential biodistribution and effective lung targeting. Finally, combined with the available human PK data,^16^ the clinical potential of TET for the treatment of SARS‐CoV‐2 infection was evaluated.

As a known blocker of the TPC, TET appears to have broad antiviral activity. It can inhibit the host cell entrance of the Ebola virus with an EC_50_ of 55 nM, HCoV‐OC43 with an EC_50_ of 0.33 µM in MRC‐5 cells, and MERS‐CoV spike protein pseudovirions.^3^, ^4^, ^17^ Previous studies reported that TET potently inhibited SARS‐CoV‐2 infection in Vero E6 cells with an EC_50_ of 2.3 µM.^8^ Our experiments consistently demonstrated that TET efficiently blocked SARS‐CoV‐2 infection (MOI = 0.05) with EC_50_ values of 2.36 µM (SI = 10.39) and 0.40 µM (SI = 36.85) in Vero E6 and Huh7 cells, respectively (Figure 1A,B). The EC_50_ and EC_90_ of TET against SARS‐CoV‐2 are higher (10.37 and 14.80 µM) in Vero E6 cells overexpressing transmembrane protease serine 2 (TMPRSS2, a co‐receptor for SARS‐CoV‐2 entry).^18^ The inhibition potency of TET was less in Calu‐3 lung cells (EC_50_ of 5.03 µM and EC_90_ of 12.08 µM) than in Vero E6 and Huh7 cells at MOI of 0.05 (Figure 1A–C). This may be due to the high expression levels of TMPRSS2 in Calu‐3 cells, leading to a partial bypass of virus entry via the endocytosis pathway.^19^ However, the EC_50_ values of TET against SARS‐CoV‐2 in the three cell lines were similar at MOI of 0.01 (Figure 1D–F). Thus, the underlying mechanism causing the difference remains to be further investigated.

Although, roughly compared with the currently approved clinical drugs, such as remdesivir (EC_50_ ≈ 0.7 µM) and molnupiravir (EC_50_ ≈ 0.3 µM), both of which directly bind to viral proteins,^20^, ^21^ the EC_50_ value of TET against SARS‐CoV‐2 was higher, TET targets host TPC1 and TPC2 and may have different antiviral mechanisms. TPC1 and TPC2 are located within the endolysosomal system, where they regulate the endolysosomal microenvironment and trafficking dynamics.^19^, ^22^ TPC blockers or genetic ablation of TPCs affects endosomal trafficking of viruses and bacterial toxins, reducing infectivity.^17^, ^23^ TPC activity alters endolysosomal Ca^2+^ content and pH, which could impair endolysosomal trafficking and the activity of proprotein convertases (such as furin) required for proteolytic activation of the spike protein in coronavirus fusion.^17^, ^24^ Ou et al.^5^ showed that inhibition of TPC2 blocks SARS‐CoV‐2 pseudovirion entry. Here, we also showed that TET mainly inhibited SARS‐CoV‐2 at the early‐entry stage by dysregulating virus trafficking among the ELs and trapping the viral particles within the intracellular EEs in Vero E6 cells (Figures 2 and 3). In addition, consistent with previous reports,^17^, ^25^ we observed that TET enlarged EEs and ELs and disrupted the regular vesicle membrane (Figure 3), which may also affect its maturation and functionality.

Further in vivo studies using the AdV5‐hACE2‐transduced mouse model showed that TET did not reduce pulmonary viral load but significantly reduced virus‐induced murine lung injury in a dose‐dependent manner (Figure 4). In severe COVID‐19, DAD is prominent, with fibrin exudates, hyaline membrane formation, thickening of the alveolar walls, and an uncontrolled inflammatory response, and up to 17% of individuals with COVID‐19 develop pulmonary fibrosis.^7^ TET is an anti‐inflammatory and immunosuppressive agent that has been used in the treatment of various pulmonary diseases.^26^ It potently inhibits the proliferation and production of cytokines and interleukins (such as IL‐5, IL‐6, tumor necrosis factor‐α, prostaglandin, and cycloxygenase‐2) and activation of T cells to reduce inflammation.^27^, ^28^ In addition, it reverses silicosis through its anti‐fibrotic activity and inhibits mitogen‐induced lymphocyte proliferation and antibody production by B cells in human myeloid leukemia cells through its immunosuppressive effects.^6^, ^29^ Kim et al.^4^ showed that TET significantly reduced the levels of inflammatory cytokines, including interferon‐λ, IL‐1β, IL‐6, and IL‐8, induced by HCoV‐OC43 infection in MRC‐5 cells. The mechanism by which TET alleviates SARS‐CoV‐2‐induced lung injury and inflammation needs further investigation. Combined with the multifunctional role of TET in treating severe lung damage, it is important to explore the clinical use of TET for the treatment of severe COVID‐19.

Although it has been reported that there is no sex difference in the PK characteristics of TET in rats,^30^ we found significant sex differences in the effects of TET in rat plasma (Figure 5). The *C*_max_ and AUC values of plasma TET in female rats were approximately twice as high as those in male rats at the same dosage and administration routes (i.v., p.o., and i.t.), which might be attributable to sex‐related differences in drug‐metabolizing enzymes in rats. Metabolic reaction phenotyping experiments showed that TET is metabolized mainly by CYP3A (Figure S3). CYP levels in female rats are 10%–30% lower than those in male rats.^31^, ^32^ However, in human populations, variability in xenobiotic metabolism is predominantly related to intra‐individual variation due to environmental exposure, rather than sex‐dependent differences in CYP3A isoforms.^33^ Therefore, in humans, TET metabolism is unlikely to differ by sex, which is why we used in vivo data and carried out an analysis based on the results from male rats.

Following oral administration, TET was slowly absorbed (*T*_max_ = 6.7 h), with an oral bioavailability (*F*) value of 33.6%. The fraction of TET oral dose absorbed (*F*_a_ × *F*_g_) in rats (36.5%) was very close to that of *F* (33.6%), and the very low hepatic blood CL and extraction rate (3.26%) were consistent with the outcomes obtained from the metabolic stability experiment in RLM, suggesting that liver metabolism might not be the primary elimination pathway of TET. The low plasma concentration might not have been induced by hepatic metabolism. Our results suggest that the extrapolated human hepatic extraction rate from HLM (3.04%) was similar to that of rats; therefore, we speculated that the PK behavior of TET in humans might be similar to that of rats. After i.v. administration of TET in rats, *V*_d_ (24.8 ± 0.952 L/kg) was much larger than the total extracellular fluid volume in rats (approximately 0.15 L/kg),^34^ suggesting that TET is widely distributed in tissues and circulation after entering the bloodstream.^35^, ^36^ The plasma concentration of TET in the elimination phase decreased slowly and fluctuated, regardless of the route of administration, probably because of the slow release of TET from tissues into the blood.

At the beginning of the experiment after oral administration, the plasma concentration of TET was far below the EC_90_ determined in the context of SARS‐CoV‐2 infection. Even with correction for the measured high *R*_b/p_ (3.08) value, the concentration of TET in whole blood remained considerably below the EC_90_. To explore the pulmonary exposure levels of TET at the target sites, the tissue distribution of TET in rats was assessed. The biodistribution results in rats showed that the concentration of TET in major tissues was much higher than that in plasma, and exposure was extremely high in the liver, spleen, lung, kidney, and intestine. Considering the physiological characteristics of these tissues and the physicochemical properties of TET, the possible mechanisms of the high distribution of TET might involve: (1) active transporters located on the membrane of these tissues facilitating the distribution of TET against the concentration gradient^37^, ^38^, ^39^; (2) TET sequestration in tissues rich in lysosomes through pH‐driven lysosomal trapping, which could lead to enormous concentrations within lysosomes^40^; or (3) TET entering monocytes/macrophage‐rich tissues with circulating monocytes in the blood.^41^ However, the mechanism needs further investigation. Regardless of the underlying mechanism, high pulmonary engagement with TET is beneficial for the treatment of lung diseases.

Although a high pulmonary distribution of TET is the desired outcome against COVID‐19, extensive exposure in other tissues, especially in the liver, may pose a risk of toxicity.^42^ Considering that administration by inhalation leads to higher pulmonary accumulation and lower liver accumulation,^43^, ^44^ we investigated the TET distribution profiles in rats after i.t. administration. The results showed that the *K*_p_ value of the lungs was increased, but the *K*_p_ values of the other tissues decreased significantly after i.t. administration compared with oral administration (Table 1). Administration of TET by inhalation could help to achieve an effective concentration of TET in the lungs and reduce the risk of toxicity in other tissues.

In this study, in vivo functional readouts of TET were evaluated in a mouse‐based SARS‐CoV‐2 infection model, while PK assessments were conducted in uninfected rats. An interspecies scaling approach based on body surface area (BSA) was used to estimate PK profiles in mice. Among the approaches for dose translation between laboratory animals, allometric scaling by BSA produces a better correlation between plasma volume/total circulating plasma protein than dosing based on either height or weight.^45^ According to the BSA method, a dose of 60 mg/kg in mice was equivalent to a dose of approximately 30 mg/kg in rats. Therefore, in this study, in vivo efficacy was bridged with PK by dose translation.

Although a robust antiviral effect is obtained when the free concentration at the target site is above the in vitro unbound EC_90_ value, the unbound plasma concentration is usually taken as a surrogate because the unbound plasma and unbound lung concentrations are assumed to be in equilibrium by rapid blood perfusion through the lung.^46^ However, as TET has an extremely high tissue distribution, the use of plasma concentrations to evaluate its antiviral effect may severely underestimate its efficacy. By taking the highest unbound EC_90_ value (worst‐case scenario of 1.52 µg/ml) as an efficacy indicator, studies on tissue distribution showed that the unbound concentration in the lung could be above unbound EC_90_ after repeated dosing of TET at 30 mg/kg through oral administration (from 0.43 µg/ml after a single dose to 2.28 µg/ml after multiple doses). This was confirmed by an in vivo efficacy study in mice receiving TET at doses above 60 mg/kg. Although, in this study, lung exposure after a single i.t. administration of 7 mg/kg was not effective (0.61 µg/ml), it has the potential to improve tissue specificity and reduce dose and toxicity risk after repeated i.t. administration due to its ability to yield higher lung exposure and lower exposure to other tissues compared to oral administration, and eventually found a new way for TET development.

Finally, the relationships between dose–route–target site exposures were investigated to anticipate human efficacy based on available PK profiles for TET repurposing. Regarding the clinical PK of TET, a reliable plasma *C*_max_ of 67.3 ng/ml after a single oral administration of 100 mg TET tablet was identified.^16^ Considering that *K*_p,uu_ is a critical parameter to bridge PK and pharmacodynamics, as well as across species extrapolation,^47^ the unbound lung concentration in humans after single oral dose of 100 mg TET would be approximately 0.24 µg/ml according to our present investigation. Currently, the clinical dosing regimen of TET for the treatment of silicosis in China is recommended as 60–100 mg/time, three times daily for 6 days, and 1 day off for 3 months.^5^, ^48^ Integrated with the tissue distribution results in rats after six consecutive days of administration, it is likely that the unbound lung concentration could be close to the effective exposure. However, our study also provides evidence that inhalation is a better route for reducing off‐target toxicity. Another limitation of this study is that, owing to biosafety restrictions, all PK studies were conducted in normal animals, and PK studies in virus‐infected mice have not been designed to obtain more direct lung exposure concentrations. This is a direction for future research.

In this study, we systemically confirmed the antiviral potency of TET in both in vitro and in vivo models. TET efficiently blocked SARS‐CoV‐2 infection in vitro and significantly alleviated virus‐induced murine lung injury, suggesting that it might have dual mechanisms of both inhibiting viral replication and attenuating inflammatory response. The plasma or whole blood concentration of TET is not a proper surrogate because of its wide distribution and retention in the lungs. After a comparison of the biodistribution and pulmonary targeting ability of TET via different administration routes to the in vitro antiviral activity against SARS‐CoV‐2, a favorable biodistribution of TET after i.t. administration suggested that inhaled administration may be a more effective route than oral dosing to treat COVID‐19. Finally, a clinical perspective of TET against SARS‐CoV‐2 is proposed. This work helps to better understand the biodistribution of TET in the respiratory tract under different administration routes, which will bridge the gap between in vitro activity and in vivo efficacy and facilitate the translational development of TET in the context of SARS‐CoV‐2 infection. The crucial pharmacodynamic and PK data obtained in this study provide useful references for further optimization of the therapeutic strategies of TET in treating COVID‐19 and other diseases.

## 4 MATERIALS AND METHODS

### 4.1 Cells and virus

Vero E6 cells were cultured in minimum Eagle's medium supplemented with 10% fetal bovine serum (FBS; Gibco Invitrogen); Huh7 and Calu‐3 cells were cultured in Dulbecco's modified Eagle's medium supplemented with 10% FBS. SARS‐CoV‐2 (nCoV‐2019BetaCoV/Wuhan/WIV04/2019 strain) was propagated in Vero E6 cells, and the virus stock was stored at −80°C and titrated by an end‐point dilution assay, as previously described.^21^ All infection experiments were performed in a biosafety level‐3 laboratory.

### 4.2 Chemicals and materials

TET (purity >99.2%; chemical structure shown in Figure S1) was supplied by Conba Pharmaceutical Co., Ltd. (Hangzhou, China). Buspirone hydrochloride (purity >99%, internal standard) and DMSO (purity >99.7%) were purchased from Sigma–Aldrich (St. Louis, MO, USA). HPLC‐grade acetonitrile (ACN) and rapid equilibrium dialysis (RED) device inserts were purchased from Thermo Fisher Scientific (Waltham, MA, USA). HPLC‐grade formic acid was obtained from Dikma Technologies Inc. (Foothill Ranch, CA, USA). An RM‐003 liquid atomizer was purchased from Raymain Information Technology Co., Ltd. (Shanghai, China). Recombinant human CYP (rCYPs) were purchased from BD Biosciences (Bedford, MA, USA). RLM and HLM were purchased from Sekisui XenoTech (Kansas City, KS, USA).

### 4.3 Animals

Eight to twelve‐week‐old SPF, female transgenic IFNAR^−/−^ C57/BL6 mice were bred in the animal center of the Wuhan Institute of Virology, CAS (WIVA01202001).

Healthy Sprague–Dawley rats (200–230 g) were purchased from the Beijing Vital River Laboratory Animal Technology Co., Ltd. The animals were housed in an appropriate environment and provided chow and water ad libitum. The rats were fasted for 12 h before oral administration of drug. All experiments were conducted at the Beijing Center for Drug Safety Evaluation after obtaining ethical approval (IACUC‐DWZX‐2021‐662) from the Institutional Animal Care and Use Committee of the Center, which followed the guidelines of the Association for Assessment and Accreditation of Laboratory Animal Care International.

### 4.4 Evaluation of TET against SARS‐CoV‐2 replication

The antiviral evaluation of the compounds in vitro was performed as previously described.^21^ First, the cytotoxicity of TET on Vero E6, Huh7, and Calu‐3 cells was detected using CCK8 kit (Beyotime, China). To determine the antiviral efficiency of the drug, monolayer cells cultured in a 48‐well cell‐culture petri dish were pre‐treated with different concentrations of TET for 1 h at 37°C and incubated with SARS‐CoV‐2 at 0.05 or 0.01 MOI for 2 h. Then, the drug–virus mixture was washed and replaced with the fresh drug‐containing medium. The supernatant of the infected cells was harvested at 24 h p.i. (Vero E6) or 48 h p.i. (Calu‐3 and Huh7), and viral RNA levels in the supernatant were quantified using qRT‐PCR.

### 4.5 Exploring the mechanism of TET in inhibition of SARS‐CoV‐2 infection

To define the step of SARS‐CoV‐2 infection targeted by TET, a time‐of‐addition assay was performed as previously described.^21^ Vero E6 cells were treated with TET (5 µM) during entry, post‐entry, or the entire infection process (full‐time) (MOI = 0.05). At 16 h p.i., the virus yield in the supernatant was quantified by qRT‐PCR. The expression of viral NP in the infected cells was analyzed by western blotting and IFA.^48^ To investigate the impact of TET on viral intracellular trafficking, a colocalization analysis of virions with endosomes was carried out as previously described.^49^ Briefly, a monolayer of Vero E6 cells cultured in the 35‐mm glass‐bottom culture dishes was pre‐treated with TET (10 µM) or DMSO and incubated with SARS‐CoV‐2 (MOI = 5) at 4°C for 1 h. Then, the supernatant was replaced with the drug‐containing medium. And the cells were cultured at 37°C and then fixed with 4% (w/v) paraformaldehyde at different time points (15, 30, 45, and 60 min p.i.). Anti‐NP, anti‐EEA1, or anti‐LAMP1 antibodies were used for immunofluorescence staining of the viral particles, EEA1^+^ EEs, and LAMP1^+^ endosomes/lysosomes, respectively. Fluorescence images were captured using a two‐photon microscope (A1RMP, Nikon). The proportion of endosome (green) colocalized virus particles (yellow) to all virus particles (red) in the cells (*n* > 150 cells) was quantified using ImageJ (Colocation Threshold plugin). Statistical significance was assessed using one‐way analysis of variance.

### 4.6 Antiviral assessment of TET in vivo

The antiviral efficiency of TET against SARS‐CoV‐2 infection in vivo was tested in AdV5‐hACE2‐transduced, aged 8–12 weeks SPF, male IFNAR^−/−^ C57/BL6 mice, as previously described.^17^ In brief, the mice were challenged with 1 × 10^6^ TCID_50_ of SARS‐CoV‐2 intranasally at 5 days post‐transduced with 2.5 × 10^8^ TCID_50_ of AdV5‐hACE2, administered intranasally. The infected mice were intraperitoneally injected with vehicle or TET (90, 60, and 30 mg/kg) daily for five consecutive days. The infected mice were observed daily and sacrificed at 5 days p.i. The lungs were subjected to viral load analysis using qRT‐PCR, titrated using an end‐point dilution assay, and histopathological analysis.

### 4.7 Pharmacokinetic study in rats via different administration routes

Preclinical PK studies of TET were conducted in rats via i.v., i.t., and p.o. administration (*n* = 6 for each group, male:female = 1:1). One group of rats was administered an i.v. injection (dissolved in 1.2% glacial acetic acid) at 5 mg/kg. Another group received intragastrically administered 30 mg/kg TET suspended in 0.5% carboxymethyl cellulose sodium (CMC‐Na) solution. The other two groups of rats were administered by i.t. administration (simulating drug delivery by inhalation) at 7 and 14 mg/kg using the RM‐003 liquid atomizer (dissolved in 22.5 mg/ml aspartic acid solution). Blood samples were collected from the jugular vein into heparin‐anticoagulant tubes when the rats were anesthetized with isoflurane at 0, 0.033 (i.v. dosing only), 0.083, 0.25, 0.5, 1, 2, 4, 6, 8, 12, 24, 36, 48, 72, 96, and 120 h according to the pilot study results. Blood samples (approximately 100 µl) were centrifuged at 2500 × *g* at 4°C for 10 min to harvest plasma within 1 h and were kept at −40°C for analysis.

### 4.8 Tissue distribution study in rats via p.o. and i.t

Biodistribution characteristics in various organs of the TET were compared via p.o. and i.t. routes. Forty‐five male Sprague–Dawley rats were randomly divided into 15 groups (*n* = 3 for each group). Five groups of rats received a single oral administration of TET (30 mg/kg) suspended in 0.5% CMC‐Na solution, five groups received repeated oral administration of TET (30 mg/kg) daily for six consecutive days, and the other five groups received a single i.t. injection of TET (7 mg/kg), as described in Section 4.7. For each route of administration, one group of rats was euthanized at the desired time points (2, 6, 24, 144, and 240 h). The major organs and tissues, including the heart, liver, spleen, lung, kidney, brain, stomach, intestine, testis, ovary, uterus, adipose, muscle, and plasma, were immediately harvested, and all the tissues were thoroughly rinsed in physiological saline. All the samples were weighed and stored at −40°C for analysis.

### 4.9 Protein binding and blood/plasma partitioning

The protein binding rates of TET in plasma, liver microsome incubate (0.5 mg/ml protein), tissue homogenates, and 2% FBS were determined using the equilibrium dialysis method according to the instructions of the RED device (details in Supporting Information).

The *R*_b/p_ of TET was determined using freshly collected rat blood 4 h after oral administration of 30 mg/kg TET (*n* = 3) to protect blood cells from damage by any organic solvent introduced during the dissolution of TET. The obtained blood samples were divided into two parts: one part was immediately centrifuged for 10 min at 2500 × *g* to harvest the plasma, and the other part was sonicated for 15 min to break the blood cells. Each sample was matrix matched and subjected to protein precipitation. The concentration of TET in the blood and plasma was determined using LC‐MS/MS. The *R*_b/p_ ratio for TET was calculated as the ratio of the TET concentration in the blood to that in the plasma.

### 4.10 Metabolic stability in liver microsomes

TET (1 µM, 1% ACN) was incubated in RLM or HLM (0.5 mg/ml), diluted in 100 mM PBS (pH 7.4) containing MgCl_2_ (3 mM). After pre‐incubation for 5 min, the reaction was initiated by adding NADPH (final concentration of 1 mM) at 37°C. Periodic aliquots of the incubation mixture were removed at 0, 5, 15, 30, and 60 min and quenched with ACN. After centrifugation, the supernatant was collected and analyzed for the depletion by LC‐MS/MS. Negative control incubations in the absence of NADPH and positive control incubations in the presence of cocktailed CYP probes were performed simultaneously. Intrinsic clearance and hepatic clearance were calculated based on a well‐stirred model.^50^

### 4.11 CYP reaction phenotyping in recombinant human CYP enzymes

The optimized experimental conditions for CYP reaction phenotyping followed the above metabolic stability studies, with linear depletion obtained after incubation for 30 min.

TET (1 µM) was incubated with a panel of recombinant human CYP enzymes (CYP1A2, CYP2A6, CYP2B6, CYP2C8, CYP2C9, CYP2C19, CYP2D6, CYP2E1, CYP2J2, CYP3A4, CYP3A5, and CYP4F2, 20 pmol/ml each) in 100 mM PBS (pH 7.4) containing NADPH (1 mM) and MgCl_2_ (3 mM) at 37°C for 30 min (*n* = 3). A negative control group using microtomes infected with the control baculovirus was used simultaneously. The reaction was then quenched with ACN. Following centrifugation, the supernatant was analyzed using LC‐MS/MS. The remaining percentage of TET in the individual rCYP isoforms was calculated by comparing the concentrations before and after incubation.

### 4.12 Data processing and statistical analysis

The PK parameters, including CL, volume of distribution (*V*_d_), *T*_1/2_, AUC_(0–_ *_t_*_)_, AUC_(0–∞),_ and mean residence time, were calculated using WinNonlin 7.0 (Pharsight, CA, USA) according to the non‐compartmental model.

Bioavailability (*F*) was calculated using Equation (1):

(1) $$F\;=\frac{\mathrm{AU}C_{\left(0-\infty ,\;\mathrm{extravascular}\right)}\;\times \;\mathrm{dos}e_{i.v.}}{\mathrm{AU}C_{\left(0-\infty ,\;i.v.\right)}\;\times \;\mathrm{dos}e_{\mathrm{extravascular}}}\;$$

The tissue partition ratio (*K*_p_) of each tissue was calculated using Equation (2):

(2) $$K_{p}=\frac{\mathrm{AU}C_{\left(0-\infty ,\;\mathrm{tissue}\right)}}{\mathrm{AU}C_{\left(0-\infty ,\;\mathrm{plasma}\right)}}\;$$

The unbound fraction of TET in plasma and tissue homogenates (*f*_u,_ *_x_*) was calculated using Equation (3):

(3) $$f_{u,\;x}\;=\frac{\mathrm{Con}c_{\mathrm{buffer}\;\mathrm{chamber}}}{\mathrm{Con}c_{\mathrm{sample}\;\mathrm{chamber}}}\;$$

The unbound fraction in tissues was adjusted by the fold of the dilution factor (*D*), as shown in Equation (4)^51^:

(4) $$\mathrm{Undiluted}\;f_{u,\;\mathrm{tissue}}=\frac{1/D}{\left(\left(1/f_{u,\;x}\right)-1\right)+1/D}$$

The unbound fraction was used to calculate the unbound drug concentration in the plasma and tissues.

The contribution of each rCYP was calculated using the TNR approach reported by Rodrigues.^52^ The relative contribution of the CYP isoforms to TET metabolism was estimated using Equation (5)^52^:

(5) $$\begin{array}{ccc} & & \mathrm{TNR}\left(\%\right)=\frac{\mathrm{NR}}{\mathrm{TNR}}\times 100\%\\ & & \;=\frac{\mathrm{pmol}/\min/\mathrm{pmol}\;\mathrm{rCYP}\;\times \;\mathrm{pmol}\;\mathrm{CYP}/\mathrm{mg}}{\sum \left(\mathrm{pmol}/\min/\mathrm{pmol}\;\mathrm{rCYP}\;\times \;\mathrm{pmol}\;\mathrm{CYP}/\mathrm{mg}\right)}\\ & & \;\times \;100\% \end{array}$$

The normalized rate (NR) was calculated as the reaction rate multiplied by the mean specific content of the corresponding CYP in the native HLM for each rCYP, which was expressed as pmol/min/mg microsomal protein.^52^

The TET depletion half‐life in microsomal incubates and apparent CL_int_ were calculated according to a well‐stirred model. The natural log of concentration versus time was fitted using linear regression, and the slope (*k*) was converted to *T*_1/2_ values, where *T*_1/2_ = −0.693/*k*. The CL_int_ was estimated using Equation (6).^53^

(6) $$\;CL_{\mathrm{int}}=\;\frac{0.693}{\mathrm{in}\;\mathrm{vitro}\;T_{1/2}}\times \frac{\mathrm{volume}\;\mathrm{of}\;\mathrm{incubation}\;\left(\mu l\right)}{\mathrm{amount}\;\mathrm{of}\;\mathrm{microsomal}\;\mathrm{protein}\;\mathrm{in}\;\mathrm{incubation}\;\left(\mathrm{mg}\right)}\times \frac{45\;\mathrm{mg}\;\mathrm{microsomes}}{g\;\mathrm{liver}}\times \frac{g\;\mathrm{liver}}{\mathrm{kg}\;\mathrm{body}\;\mathrm{weight}}$$

The CL_h,blood_ was calculated according to Equation (7), using a hepatic blood flow (*Q*) value of 55.2 ml/min/kg for rats, and 20.7 ml/min/kg for humans.^34^ The *f*_u,p_, *f*_u,mic_, and *R*_b/p_ were obtained from measured values.

(7) $$\;{\mathrm{CL}}_{h}=\frac{Q\;\times \;f_{u,\;p}/R_{b/p}\times \;{\mathrm{CL}}_{\mathrm{int}}\;/\;f_{u,\mathrm{mic}}}{Q\;+f_{u,\;p}/R_{b/p}\times \;{\mathrm{CL}}_{\mathrm{int}}\;/\;f_{u,\;\mathrm{mic}}}$$

The fraction of absorbed oral dose (*F*_a_ × *F*_g_) was estimated using Equation (8):

(8) $$F_{a}\times F_{g}=\;\frac{F}{1-CL_{\mathrm{blood}}/Q}$$

The appropriate statistical analysis was performed for each experiment. In the statistical analyses, *t*‐tests were used to compare the results of the control and treated groups. The statistical methods and statistical significance are described in the figure legends.

## AUTHOR CONTRIBUTIONS

W.Z., M.W., X.Z., and R.C. designed the experiments and contributed to the review of the manuscript. J.L., F.W., X.W., and S.F. carried out the experiments, the data processing, and information analysis and wrote the manuscript. H.H., K.L., B.Z., W.Z., M.X., L.W., H.Z., J.L., W.L., and Y.L. assisted the experiments. W.Z., M.W., X.Z., and Z.H. administrated the project. All authors have read and approved the final manuscript.

## CONFLICT OF INTEREST

The authors declare they have no conflicts of interests.

## ETHICS STATEMENT

All the experiments were conducted in Beijing Center for Drug Safety Evaluation after obtaining an ethical approval (IACUC‐DWZX‐2021‐662) from the Institutional Animal Care and Use Committee of the Centre, which followed the guidelines of the Association for Assessment and Accreditation of Laboratory Animal Care International (AAALAC).

## Supporting information

Supporting Information

## Data Availability

All data are available from the corresponding authors upon request.
