# Supplementary material for: Antiviral effects and tissue exposure of tetrandrine against SARS‐CoV‐2 infection and COVID‐19
Source: MedComm (2020). 2023 Jan 19;4(1):e206. doi: 10.1002/mco2.206 (PMC9851407; doi:10.1002/mco2.206)
Supplement: Supplementary file 1 — Supporting Information [file MCO2-4-e206-s001.docx]

**Title page**

**Supplementary Materials**

**Antiviral effects and tissue exposure of tetrandrine against SARS-CoV-2 infection and COVID-19**

**Jia Liu^b,^ ^#^, Furun Wang^a,^ ^#^, Xi Wang^b,^ ^#^, Shiyong Fan^a,^ ^#^, Yufeng Li^b^, Mingyue Xu^b^, Hengrui Hu^b^, Ke Liu^a^, Bohong Zheng^a^, Lingchao Wang^a^, Huanyu Zhang^b^, Jiang Li^b^, Wei Li^a^, Wenpeng Zhang^a^, Zhihong Hu^b^, Ruiyuan Cao^a,^*, Xiaomei Zhuang^a,^*, Manli Wang^b,^*, Wu Zhong^a,^***

^a^ National Engineering Research Center for the Emergency Drug, Beijing Institute of Pharmacology and Toxicology, Beijing 100850, China

^b^ State Key Laboratory of Virology, Wuhan Institute of Virology, Center for Biosafety Mega-Science, Chinese Academy of Sciences, Wuhan 430071, China

† These authors contributed equally to this work.

*Corresponding authors. Tel.: +86 15810563797; +86 13683600728; +86 13501334184.

E-mail addresses: 21cc@163.com (Ruiyuan Cao), xiaomeizhuang@163.com (Xiaomei Zhuang), wangml@wh.iov.cn (Manli Wang), zhongwu@bmi.ac.cn(Wu Zhong).

**Protein binding and blood/plasma partitioning**

200 μL of different biometrics spiked with 5 μM TET (0.1% DMSO) was added into the donor chamber, while 400 μL phosphate-buffered saline (PBS, pH 7.4) was added into the receiver chamber (n = 3). The RED device plate was sealed with a gas-permeable membrane and incubated at 37 °C for 5 h before all the samples were matrix-matched and quenched by protein precipitation, followed by an analysis of TET using LC-MS/MS. A set of satellite groups was included to assess the stability following 5 h of incubation. The unbound fraction of TET was calculated by dividing the concentration in the receiver sample by the concentration in the donor sample, corrected by dilution factors of tissue homogenates.

**Bioanalytical methods and validation**

All the samples mentioned above were precipitated with acetonitrile (containing IS, buspirone, 5ng/mL) and analyzed with an API 5000 Triple quadrupole mass spectrometer (AB Sciex, United States) connected to a Shimadzu LC-20AD HPLC system (Shimadzu, Japan). The chromatographic column was C18 column (3.0 mm × 50 mm, 2.6 µm, Phenomenex). The mobile phase consisted of water containing 0.1% of formic acid (A) and acetonitrile containing 0.1% of formic acid (B). Separation was achieved following a binary gradient elution procedure: 0-0.3 min B 10%, 0.3-1.4 min B 10%→90%, 1.4-1.8 min B 90%, 1.8-1.9 min B 90%→10%, 1.9-3.0 min B 10%. The volume of each injection was 5.0 µL, and the flow rate was 0.6 mL/min.

TET and IS were detected by multiple reaction monitoring (MRM) in the positive ion mode. The precursor and product ions used for quantification were as follows: *m/z* 609.0→381.1 for TET and *m/z* 386.4→122.1 for IS, respectively. Retention times were 1.31 and 1.41 minutes for TET and IS, respectively.

The bioanalytical methods of plasma and twelve tissues were fully validated according to the ICH guideline M10 on bioanalytical method validation. The evaluation indicators included selectivity, specificity, linearity, matrix effect, accuracy and precision, extraction recovery, and stability of TET in the investigated matrix.


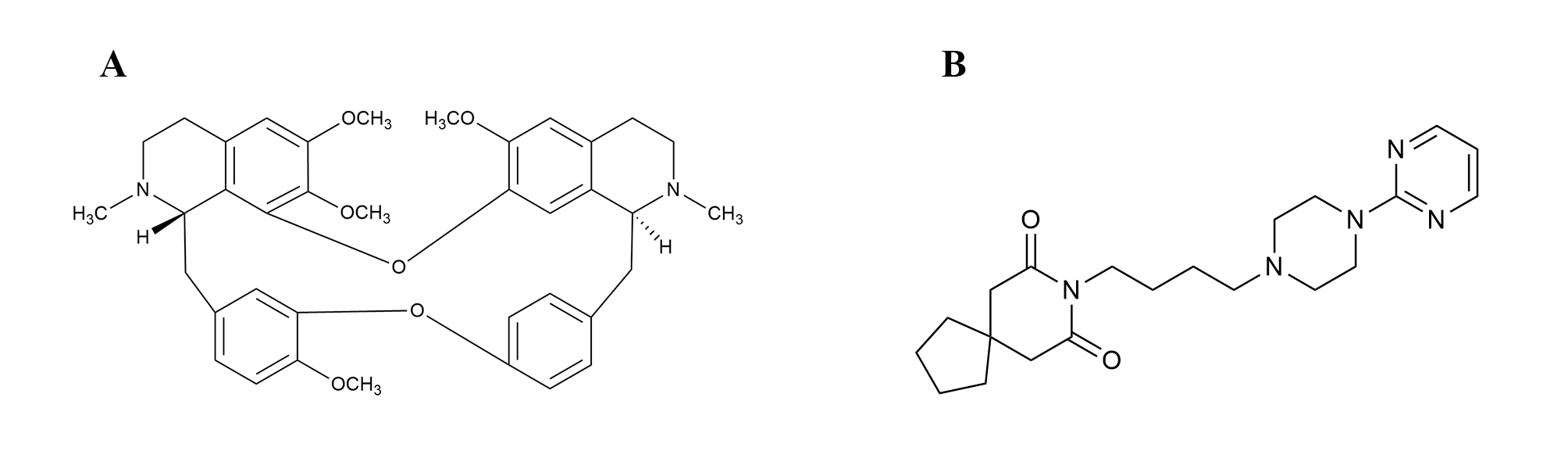


**Figure S1** The chemical structures of TET (A) and buspirone (B).


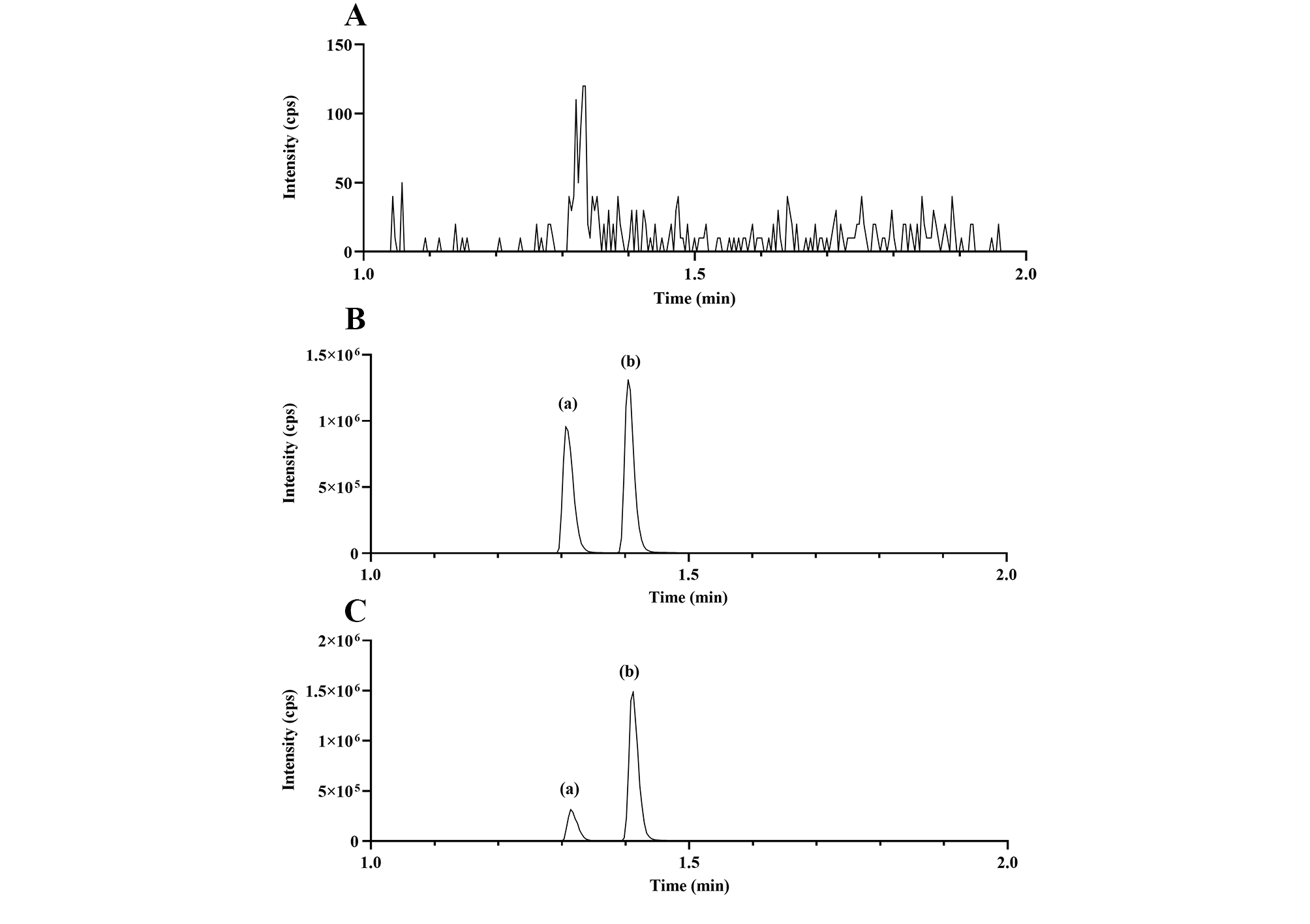


**Figure S2** The chromatogram peaks of TET (a) and buspirone (b). (A) TET in blank rat plasma, (B) plasma spiked with working solution, and (C) plasma after the inhalation of TET (7 mg/kg) in rats.

**
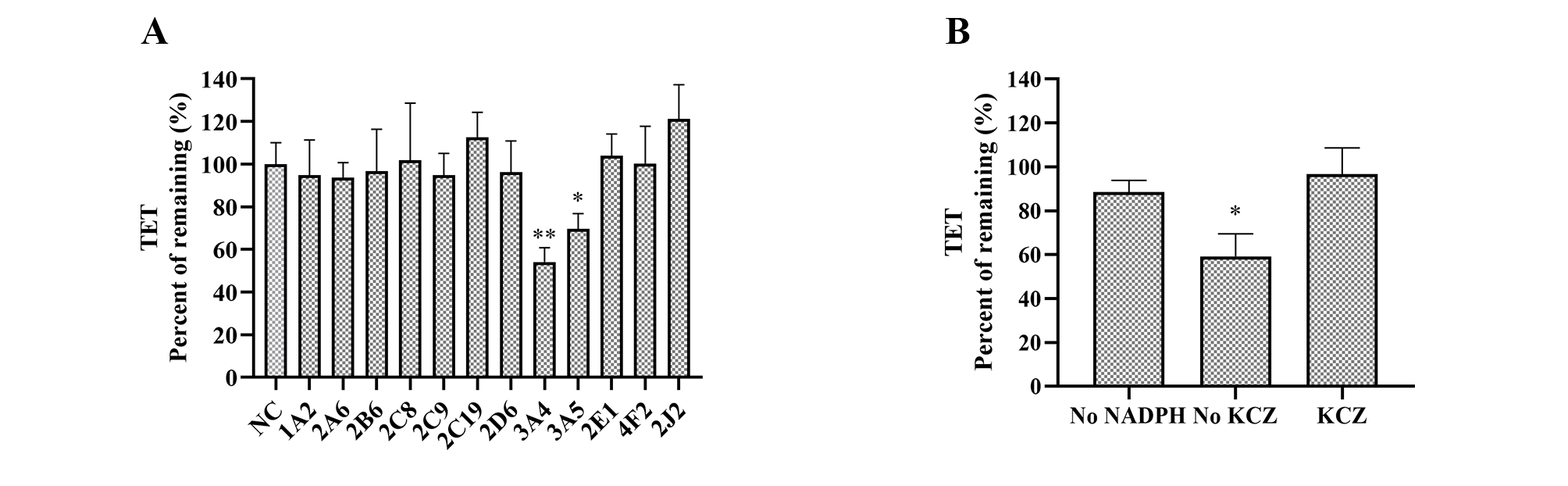
**

**Figure S3** The contribution of CYP 3A in the metabolic depletion of TET. (A) TET (1 μM) was incubated in a panel of recombinant human CYP enzymes (CYP1A2, CYP2A6, CYP2B6, CYP2C8, CYP2C9, CYP2C19, CYP2D6, CYP2E1, CYP2J2, CYP3A4, CYP3A5, and CYP4F2, 20 pmol/mL each) in 100 mM PBS (pH 7.4) containing NADPH (1 mM) and MgCl_2_ (3 mM) at 37 °C for 30 min. Negative control (NC) group using control supersomes expressed in Sf9 insect cells (without P450 expression) was conducted simultaneously (n = 3). (B) TET (1 μM) was incubated in HLM (0.5 mg/mL protein) in 100 mM PBS (pH 7.4) containing NADPH (1 mM) and MgCl_2_ (3 mM) at 37 °C for 30 min with or without ketoconazole (KCZ, 1 μM). Negative control group without NADPH was conducted simultaneously (n = 3, Mean ± SD). * indicates P < 0.05, ** indicates P < 0.01 compared to NC by t-test.

**Table S1** Accuracy and precision of TET in rat plasma and tissues (n = 6, Mean ± SD).

| Matrix | Concentration | Intra-day (n = 6) | | | | | |  | Inter-day (n = 18) | |
| --- | --- | --- | --- | --- | --- | --- | --- | --- | --- | --- |
|  | （ng/mL） | RE% | | | RSD% | | |  | RE% | RSD% |
| Plasma | 2 | 17 | 1.9 | 11 | 15 | 14 | 3.6 |  | 10 | 13 |
|  | 4 | 0 | -9.4 | 14 | 15 | 12 | 5.5 |  | 1.4 | 14 |
|  | 80 | 6.4 | 7.4 | 9 | 3.6 | 6 | 5.8 |  | 7.6 | 5 |
|  | 1600 | 14 | 3.9 | 7.5 | 5.5 | 2.3 | 7 |  | 8.5 | 6.1 |
| Heart | 5 | -9.2 | 12 | -8.2 | 10 | 5.2 | 18 |  | -1.9 | 15 |
|  | 15 | 13 | -1.6 | 2.4 | 19 | 2.8 | 8.5 |  | 4.6 | 14 |
|  | 150 | 2.1 | -3.2 | -0.22 | 4.8 | 3.2 | 8.2 |  | -0.44 | 5.9 |
|  | 1600 | -0.63 | 4.5 | -10 | 1.3 | 2.0 | 7.7 |  | -2.1 | 7.5 |
| Liver | 5 | 0.17 | 12 | 10 | 14 | 15 | 9.5 |  | 7.5 | 13 |
|  | 15 | 2.0 | 10 | 1.0 | 7.9 | 4.8 | 4.6 |  | 4.4 | 6.9 |
|  | 150 | 9.0 | 11 | -2.1 | 3.3 | 3.6 | 2.3 |  | 6.0 | 6.4 |
|  | 1600 | -4.7 | -1.0 | -3.2 | 6.0 | 6.0 | 1.5 |  | -3.0 | 4.9 |
| Spleen | 5 | -1.7 | 14 | -9.1 | 3 | 6.8 | 15 |  | 1.0 | 13 |
|  | 15 | 3.9 | -4.0 | 10 | 3.5 | 4.6 | 8.2 |  | 3.3 | 8.0 |
|  | 150 | 6.2 | -4.7 | 6.0 | 3.1 | 5.4 | 7.1 |  | 2.5 | 7.2 |
|  | 1600 | 2.3 | -5.5 | -14 | 4.6 | 1.5 | 4.7 |  | -5.8 | 8.3 |
| Lungs | 5 | 4.4 | 1.9 | 3.7 | 9.0 | 3.6 | 15 |  | 3.3 | 9.6 |
|  | 15 | -0.60 | 0.70 | -9.4 | 7.6 | 5.1 | 4.2 |  | -3.1 | 7.3 |
|  | 150 | -3.9 | -1.8 | -4.8 | 6.9 | 1.9 | 5.5 |  | -3.5 | 5.0 |
|  | 1600 | -11 | -3.1 | 0.80 | 8.1 | 3.4 | 6.1 |  | -4.4 | 7.8 |
| Kidneys | 5 | 6.8 | 2.8 | -8.0 | 14 | 9.9 | 10 |  | 0.5 | 13 |
|  | 15 | -13 | -3.0 | 7.8 | 6.9 | 4.1 | 9.0 |  | -2.6 | 11 |
|  | 150 | -14 | -2.3 | 12 | 9.9 | 4.0 | 3.9 |  | -1.5 | 13 |
|  | 1600 | -15 | -9.4 | -14 | 6.5 | 1.7 | 9.4 |  | -13 | 6.7 |
| Brain | 5 | 18 | 3.1 | -10 | 4.2 | 15 | 14 |  | 3.5 | 16 |
|  | 15 | 11 | -1.6 | 11 | 13 | 8.5 | 4.4 |  | 6.9 | 10 |
|  | 150 | 7.4 | -8.6 | 13 | 3.1 | 7.6 | 4.7 |  | 3.8 | 10 |
|  | 1600 | -4.8 | -12 | -9.5 | 2.6 | 4.0 | 2.1 |  | -8.6 | 4.2 |
| Stomach | 5 | 15 | 0.0 | -9.0 | 20 | 9.2 | 18 |  | 1.9 | 19 |
|  | 15 | 4.2 | -2.70 | 11 | 9.4 | 2.7 | 8.9 |  | 4.3 | 9.3 |
|  | 150 | -1.1 | 3.7 | 12 | 2.2 | 1.1 | 9.2 |  | 4.9 | 7.7 |
|  | 1600 | -8.2 | -1.0 | 9.4 | 5 | 1.9 | 12.4 |  | 0.0 | 11 |
| Intestine | 5 | 15 | 16 | 2.8 | 17 | 6.5 | 19 |  | 11 | 15 |
|  | 15 | -3.6 | 0.60 | 15 | 6.7 | 4.3 | 6.0 |  | 3.9 | 9.5 |
|  | 150 | 1.8 | -5.1 | 13 | 3.1 | 2.4 | 1.1 |  | 3.2 | 7.7 |
|  | 1600 | -5.3 | -4.4 | 6.0 | 3.8 | 1.9 | 4.3 |  | -1.2 | 6.3 |
| Adipose | 5 | -0.60 | 15 | -1.4 | 7.9 | 5.1 | 18 |  | 4.2 | 13 |
|  | 15 | 1.2 | -5.6 | 13 | 5.1 | 4.5 | 6.2 |  | 3.0 | 9.3 |
|  | 150 | 4.4 | 0.70 | 14 | 3.6 | 5.8 | 6.3 |  | 6.2 | 7.3 |
|  | 1600 | -2.2 | 0.60 | -15 | 2.0 | 3.3 | 3.6 |  | -5.6 | 8.0 |
| Muscle | 5 | 13 | 9.6 | 3.3 | 14 | 17 | 17 |  | 7.6 | 15 |
|  | 15 | -12 | -1.8 | 12 | 3.6 | 3.3 | 9.0 |  | -0.63 | 12 |
|  | 150 | 1.3 | 4.9 | 12 | 6.2 | 4.3 | 7.4 |  | 6.2 | 7.3 |
|  | 1600 | -8.8 | 2.2 | -5.5 | 3.5 | 3.0 | 4.8 |  | -4.0 | 6.1 |
| Testes | 5 | 8.9 | 8.8 | 13 | 13 | 8.0 | 10 |  | 10 | 10 |
|  | 15 | 3.3 | 1.7 | 4.0 | 8.5 | 2.2 | 7.7 |  | 3.0 | 6.4 |
|  | 150 | 7.1 | -3.2 | 0.30 | 5.7 | 3.4 | 5.4 |  | 1.4 | 6.4 |
|  | 1600 | -5.2 | -11 | -8.1 | 1.5 | 4.0 | 3.4 |  | -8.0 | 3.8 |
| Uterus | 5 | 20 | 12 | 12 | 9.4 | 5.5 | 5.9 |  | 15 | 7.9 |
| & | 15 | 1.0 | 8.3 | 7.4 | 5.5 | 8.2 | 5.1 |  | 5.6 | 6.8 |
| Ovaries | 150 | 1.1 | -1.6 | -0.90 | 5.0 | 3.5 | 3.3 |  | -0.40 | 4.0 |
|  | 1600 | 1.5 | -8.9 | -6.1 | 4.1 | 1.5 | 3.5 |  | -4.5 | 5.6 |

**Table S2** Stability of TET in rat plasma and tissues under different storage conditions (n = 6, Mean ± SD).

| Matrix | Concentration （ng/mL） | Room temperature, 24h | | In autosampler, 24h | | Freeze-thaw, three times | | -40 °C,  30d | | |
| --- | --- | --- | --- | --- | --- | --- | --- | --- | --- | --- |
|  |  | RE  % | RSD  % | RE  % | RSD  % | RE  % | RSD  % | RE  % | RSD  % | |
| Plasma | 4 | -14 | 11 | 14 | 6.9 | 6.8 | 10 | 4.3 | | 15 |
|  | 80 | 10 | 6.6 | 10 | 5.6 | 4.0 | 3.0 | 10 | | 9.3 |
|  | 1600 | 4.8 | 1.2 | 6.7 | 8.5 | 10 | 5.1 | 14 | | 6.8 |
| Heart | 15 | -1.1 | 4.4 | -2.0 | 0.0 | 2.0 | 3.5 | 2.2 | | 5.5 |
|  | 150 | -3.3 | 5.0 | -3.1 | 0.80 | -1.8 | 3.4 | 1.3 | | 2.0 |
|  | 1600 | 5.0 | 2.7 | 4.0 | 1.3 | -3.8 | 1.9 | -2.7 | | 1.5 |
| Liver | 15 | 7.3 | 6.3 | -3.3 | 5.6 | 3.1 | 2.6 | -1.1 | | 5.7 |
|  | 150 | 9.3 | 4.8 | 8.7 | 1.6 | -1.6 | 2.8 | -2.7 | | 2.1 |
|  | 1600 | -1.0 | 1.3 | -8.3 | 7 | -2.9 | 1.9 | -3.5 | | 1.3 |
| Spleen | 15 | 6.0 | 2.3 | 1.8 | 3.6 | -6.0 | 4.4 | -3.8 | | 2.0 |
|  | 150 | 6.2 | 1.0 | 6.2 | 4.9 | -6.0 | 6.1 | -4.4 | | 4.5 |
|  | 1600 | 4.6 | 4.4 | 0.0 | 4.4 | -4.6 | 1.5 | -5.6 | | 1.8 |
| Lungs | 15 | -4.2 | 7.4 | 3.1 | 7.1 | -8.7 | 3.9 | -10 | | 5.3 |
|  | 150 | -7.6 | 8.8 | -0.20 | 2.0 | -4.4 | 7.4 | -5.1 | | 4.5 |
|  | 1600 | -9.0 | 9.4 | -13 | 7.6 | 2.1 | 6.2 | -0.40 | | 7.0 |
| Kidneys | 15 | 3.1 | 2.6 | 12 | 11 | -1.3 | 4.7 | -4.7 | | 3.2 |
|  | 150 | 11 | 4.9 | 13 | 3.5 | -1.8 | 4.8 | -2.9 | | 3.9 |
|  | 1600 | -13 | 6.6 | -14 | 13 | -8.8 | 2.4 | -10 | | 0.70 |
| Brain | 15 | 7.3 | 2.2 | 4.7 | 4.2 | 0.20 | 9.8 | -3.3 | | 8.5 |
|  | 150 | 8.2 | 4.1 | 7.3 | 1.6 | -8.2 | 11 | -8.9 | | 5.2 |
|  | 1600 | -4.0 | 2.5 | -6.5 | 1.4 | -10 | 5.7 | -13 | | 0.40 |
| Stomach | 15 | -1.3 | 1.8 | -4.0 | 3.0 | 6.9 | 9.9 | -0.20 | | 11 |
|  | 150 | 4.0 | 0.0 | 3.3 | 1.7 | -1.1 | 3.0 | -2.7 | | 1.2 |
|  | 1600 | -0.80 | 1.8 | -1.3 | 2.3 | -9.4 | 7.5 | -7.1 | | 2.2 |
| Intestine | 15 | 2.0 | 3.6 | -0.90 | 5.1 | -2.9 | 2.6 | 2.0 | | 6.5 |
|  | 150 | -4.2 | 2.8 | -6.0 | 1.9 | 2.4 | 3.7 | 4.9 | | 3.5 |
|  | 1600 | -5.0 | 2.3 | -3.8 | 1.7 | -4.8 | 2.0 | 8.8 | | 3.4 |
| Adipose | 15 | -3.6 | 5.3 | 11 | 5.4 | -7.6 | 2.7 | -6.0 | | 1.2 |
|  | 150 | -0.4 | 8.0 | 12 | 6.2 | 1.8 | 4.2 | 7.6 | | 1.4 |
|  | 1600 | 1.3 | 4.3 | 15 | 1.1 | 0.0 | 2.9 | 10 | | 0.30 |
| Muscle | 15 | -0.20 | 3.7 | -3.3 | 2.5 | 7.6 | 9.3 | 2.9 | | 12 |
|  | 150 | 6.9 | 1.6 | 2.9 | 5.9 | 9.6 | 2.3 | 2.2 | | 9.5 |
|  | 1600 | 1.5 | 1.5 | 2.9 | 4.3 | -7.9 | 2.4 | -4.0 | | 5.6 |
| Testes | 15 | -0.20 | 5.6 | 6.9 | 10 | 2.9 | 1.0 | 0.40 | | 2.7 |
|  | 150 | 7.1 | 8.8 | 7.1 | 1.6 | -3.6 | 2.8 | -2.9 | | 4.5 |
|  | 1600 | -4.0 | 0.40 | -6.5 | 0.80 | -10 | 4.9 | -11 | | 3.9 |
| Uterus& | 15 | 3.3 | 6.2 | -1.3 | 4.4 | -2.9 | 5.2 | 11 | | 3.9 |
| Ovaries | 150 | 2.0 | 7.2 | 0.20 | 2.7 | 0.90 | 1.5 | -3.8 | | 3.6 |
|  | 1600 | 4.8 | 2.4 | -1.9 | 1.7 | -3.3 | 6.0 | -9.4 | | 1.2 |

**Table S3** Matrix effect and recovery of TET (n = 6, Mean ± SD).

| Matrix | Concentration  （ng/mL) | Extraction  recovery (%) | Matrix effect  (%) | Matrix effect  RSD (%) |
| --- | --- | --- | --- | --- |
| Plasma | 4 | 96 ± 17 | 123 ± 14 | 11 |
|  | 80 | 94 ± 8.8 | — | — |
|  | 1600 | 93 ± 5.0 | 132 ± 4.2 | 3.2 |
| Heart | 15 | 95 ± 5.0 | 47 ± 2.5 | 5.3 |
|  | 150 | 101 ± 6.0 | — | — |
|  | 1600 | 96 ± 6.1 | 92 ± 5.9 | 6.3 |
| Liver | 15 | 115 ± 14 | 79 ± 9.9 | 12 |
|  | 150 | 89 ± 5.1 | — | — |
|  | 1600 | 83 ± 4.0 | 94 ± 4.5 | 4.7 |
| Spleen | 15 | 83 ± 5.4 | 81 ± 5.2 | 6.4 |
|  | 150 | 93 ± 2.3 | — | — |
|  | 1600 | 87 ± 3.2 | 86 ± 3.2 | 3.7 |
| Lungs | 15 | 72 ± 3.9 | 83 ± 4.5 | 5.5 |
|  | 150 | 74 ± 4.6 | — | — |
|  | 1600 | 78 ± 6.1 | 76 ± 6.0 | 7.8 |
| Kidneys | 15 | 83 ± 3.3 | 100 ± 4.1 | 4.1 |
|  | 150 | 95 ± 3.5 | — | — |
|  | 1600 | 90 ± 4.3 | 102 ± 4.9 | 4.8 |
| Brain | 15 | 93 ± 6.6 | 121 ± 8.6 | 7.1 |
|  | 150 | 93 ± 3.9 | — | — |
|  | 1600 | 94 ± 3.9 | 89 ± 3.7 | 4.2 |
| Stomach | 15 | 105 ± 11 | 44 ± 4.8 | 11 |
|  | 150 | 130 ± 4.2 | — | — |
|  | 1600 | 127 ± 5.1 | 136 ± 5.4 | 4.0 |
| Intestine | 15 | 80 ± 3.0 | 97 ± 3.6 | 3.7 |
|  | 150 | 97 ± 2.9 | — | — |
|  | 1600 | 93 ± 3.6 | 79 ± 3.0 | 3.8 |
| Adipose | 15 | 97 ± 3.3 | 94 ± 3.2 | 3.4 |
|  | 150 | 102 ± 6.3 | — | — |
|  | 1600 | 97 ± 3.0 | 83 ± 2.6 | 3.1 |
| Muscle | 15 | 96 ± 4.6 | 93 ± 4.5 | 4.8 |
|  | 150 | 99 ± 3.5 | — | — |
|  | 1600 | 100 ± 2.8 | 103 ± 2.9 | 2.8 |
| Testes | 15 | 86 ± 1.6 | 66 ± 1.2 | 1.9 |
|  | 150 | 91 ± 6.0 | — | — |
|  | 1600 | 89 ± 4.6 | 74 ± 3.9 | 5.2 |
| Uterus | 15 | 93 ± 4.4 | 61 ± 2.9 | 4.7 |
| & | 150 | 91 ± 4.2 | — | — |
| Ovaries | 1600 | 93 ± 3.8 | 106 ± 4.3 | 4.0 |

**Table S4** Pharmacokinetic parameters of TET in male rats via different administration routes. Pharmacokinetic parameters were calculated from plasma concentration-time data and are reported as mean ± S.D. (n = 3). Intravenous (*i.v.*) doses for TET were dissolved in 1.2% glacial acetic acid. Oral (*p.o.*) pharmacokinetics studies were conducted in the fast state administered with TET suspended in 0.5% carboxymethyl cellulose sodium (CMC-Na) solution. Inhaled (*i.t.*) doses were administered using the RM-003 liquid atomizer with TET dissolved in 22.5 mg/mL aspartic acid solution (n = 3, Mean ± SD).

| Parameters | Unit | *i.v.* | *p.o.* | *i.t.* | |
| --- | --- | --- | --- | --- | --- |
|  |  | 5 mg/kg | 30 mg/kg | 7 mg/kg | 14 mg/kg |
| T_1/2_ | h | 21.1 ± 1.36 | 45.9 ± 23.4 | 40.7 ± 4.72 | 38.2 ± 4.23 |
| T_max_ | h | — | 6.67 ± 4.62 | 0.139 ± 0.096 | 0.083 ± 0 |
| C_max_ | ng/mL | — | 169 ± 61.1 | 450 ± 78.7 | 1293 ± 168 |
| C_0_ | ng/mL | 525 ± 126 | — | — | — |
| AUC_(0-t)_ | h·ng/mL | 5942 ± 537 | 8992 ± 2602 | 2968 ± 286 | 6452 ± 1465 |
| AUC_(0-∞)_ | h·ng/mL | 6166 ± 573 | 12444 ± 6681 | 3411 ± 297 | 7391 ± 2357 |
| MRT_(0-t)_ | h | 33.7 ± 1.42 | 84.4 ± 41.8 | 66.7 ± 11.1 | 58.8 ± 18.3 |
| V_d_ | L/kg | 24.8 ± 0.952 | — | 26.4 ± 2.79 | — |
| CL | mL/h/kg | 815 ± 72.1 | — | 537 ± 93.3 | — |
| F | % | — | 33.6 | 39.5 | 42.8 |

**Table S5** Protein bindings of TET in different biometrics. The protein binding of TET in plasma, liver microsomes incubate (0.5 mg/mL protein), various tissue homogenates, and 2% fetal bovine serum (FBS) were determined using the equilibrium dialysis method according to the instructions of the RED device (n = 3, Mean ± SD).

| Biomatrix | | Protein binding (%) |
| --- | --- | --- |
| Plasma | Rat | 98.32 ± 0.22 |
|  | Dog | 99.32 ± 0.05 |
|  | Human | 99.23 ± 0.16 |
| Tissue | Heart | 99.87 ± 0.83 |
|  | Liver | 99.91 ± 0.32 |
|  | Spleen | 99.83 ± 1.3 |
|  | Lungs | 99.20 ± 0.01 |
|  | kidneys | 99.87 ± 1.4 |
|  | Brain | 99.92 ± 0.35 |
|  | Stomach | 99.81 ± 0.74 |
|  | Intestine | 99.99 ± 0.05 |
|  | Muscle | 99.61 ± 0.21 |
|  | Testes | 99.86 ± 0.20 |
|  | Uterus & Ovaries | 99.77 ± 0.74 |
| FBS | 2% FBS | 79.79 ± 2.6 |

**Table S6** Unbound tissue partition coefficient (K_p,uu_) of TET after different administration patterns. Unbound partition coefficient (K_p,uu_) of TET in different tissues was obtained from individual K_p_ values (Table 1) multiplied by the corresponding ratio of f_u_ in tissue and in plasma (Table S5) (n = 3, Mean ± SD).

| Tissue | *i.t.*  (Single dosing) | *p.o.*  (Single dosing) | *p.o.*  (Repeated dosing) |
| --- | --- | --- | --- |
| Lung | 315 ± 95.1 | 245 ± 124 | 299 ± 112 |
| Spleen | 72.1 ± 21.2 | 94.5 ± 17.4 | 115 ± 32.3 |
| Kidney | 31.3 ± 9.84 | 44.8 ± 12.5 | 49.8 ± 22.8 |
| Liver | 23.8 ± 12.5 | 37.1 ± 15.5 | 65.0 ± 20.1 |
| Stomach | 4.72 ± 1.68 | 5.53 ± 2.08 | 7.65 ± 1.74 |
| Testis | 8.10 ± 2.49 | 16.1 ± 11.6 | 10.0 ± 1.16 |
| Heart | 3.80 ± 0.932 | 5.11 ± 2.23 | 4.95 ± 1.81 |
| Muscle | 6.22 ± 2.31 | 8.20 ± 3.34 | 10.7 ± 6.78 |
| Intestine | 0.538 ± 0.195 | 2.57 ± 1.29 | 0.490 ± 0.114 |
| Brain | 0.837 ± 0.208 | 1.27 ± 0.409 | 0.667 ± 0.712 |
| Adipose | 0.283 ± 0.006 | 0.282 ± 0.108 | 0.392 ± 0.165 |

**Table S7** Pharmacokinetic parameters of TET in tissues and plasma after *p.o.* administration of 30 mg/kg TET in male rats. (n = 3, Mean ± SD).

| Parameters | Unit | Tissues | | | | | | | | | | | |
| --- | --- | --- | --- | --- | --- | --- | --- | --- | --- | --- | --- | --- | --- |
|  |  | Spleen | Liver | Kidney | Lung | Intestine | Testis | Heart | Stomach | Muscle | Brain | Adipose | Plasma |
| t_1/2_ | h | 29.5 ± 6.80 | 22.1 ± 3.43 | 24.5 ± 4.16 | 26.1 ± 5.00 | 24.6 ± 3.71 | 129 ± 91.6 | 28.5 ± 3.90 | 24.7 ± 7.51 | 27.8 ± 1.55 | 42.3 ± 20.9 | 35.6 ± 5.77 | 48.5 ± 11.4 |
| T_max_ | h | 6.00 ± 0 | 3.33 ± 2.31 | 6.00 ± 0 | 24.0 ± 0 | 2.00 ± 0 | 96.0 ± 124 | 6.00 ± 0 | 3.33 ± 2.31 | 12.0 ± 10.3 | 12.0 ± 10.3 | 18.0 ± 10.3 | 10.7 ± 11.7 |
| C_max_ | μg/mL | 92.6 ± 7.23 | 153 ± 42.1 | 69.8 ± 7.77 | 53.5 ± 10.9 | 244 ± 24.0 | 4.65 ± 0.630 | 9.25 ± 1.21 | 28.6 ± 33.6 | 4.46 ± 0.390 | 2.91 ± 0.130 | 1.16 ± 0.440 | 0.180 ± 0.040 |
| AUC_(0-t)_ | h·μg/mL | 7561 ± 906 | 5695 ± 840 | 4815 ± 71.8 | 4182 ± 967 | 3029 ± 596 | 755 ± 108 | 539 ± 49.2 | 440 ± 169 | 298 ± 58.5 | 233 ± 24.6 | 80.8 ± 9.07 | 9.58 ± 2.10 |
| AUC_(0-∞)_ | h·μg/mL | 7817 ± 987 | 5727 ± 838 | 4870 ± 82.4 | 4273 ± 992 | 3049 ± 592 | 1511 ± 686 | 553 ± 54.8 | 449 ± 173 | 303 ± 56.7 | 242 ± 30.7 | 82.1 ± 10.5 | 9.85 ± 2.02 |
| K_p_ | / | 789 | 594 | 502 | 437 | 316 | 78.8 | 56.3 | 45.9 | 31.1 | 24.3 | 8.43 | 1 |

**Table S8** Pharmacokinetic parameters of TET in tissues and plasma after *i.t.* administration of 7 mg/kg TET in male rats. (n = 3, Mean ± SD).

| Parameters | Unit | Tissues | | | | | | | | | | | |
| --- | --- | --- | --- | --- | --- | --- | --- | --- | --- | --- | --- | --- | --- |
|  |  | Spleen | Lung | Liver | Kidney | Testis | Intestine | Heart | Stomach | Muscle | Brain | Adipose | Plasma |
| t_1/2_ | h | 45.1 ± 6.93 | 40.7 ± 1.80 | 35.4 ± 9.41 | 40.3 ± 5.71 | 319 ± 199 | 33.6 ± 17.1 | 51.1 ± 10.5 | 25.2 ± 2.15 | 15.7 ± 0.240 | 28.9 ± 3.53 | 33.9 ± 4.47 | 53.0 ± 22.1 |
| T_max_ | h | 12.0 ± 10.4 | 4.67 ± 2.31 | 12.0 ± 10.4 | 4.67 ± 2.31 | 64.0 ± 69.3 | 3.33 ± 2.31 | 4.67 ± 2.31 | 3.33 ± 2.31 | 6.00 ± 0 | 4.67 ± 2.31 | 12.0 ± 10.4 | 6.67 ± 4.62 |
| C_max_ | μg/mL | 64.5 ± 4.77 | 76.2 ± 5.51 | 54.6 ± 11.3 | 70.8 ± 15.5 | 4.81 ± 2.26 | 35.2 ± 16.8 | 8.60 ± 3.84 | 10.5 ± 3.04 | 3.87 ± 0.900 | 3.74 ± 2.80 | 1.13 ± 0.240 | 0.169 ± 0.611 |
| AUC_(0-t)_ | h·μg/mL | 5517 ± 584 | 5286 ± 1379 | 3613 ± 1747 | 3150 ± 209 | 771 ± 68.0 | 693 ± 18.6 | 383 ± 49.0 | 326 ± 9.19 | 208 ± 5.16 | 143 ± 27.0 | 82.7 ± 32.9 | 8.99 ± 2.60 |
| AUC_(0-∞)_ | h·μg/mL | 5676 ± 604 | 5381 ± 1387 | 3638 ± 1734 | 3204 ± 224 | 1869 ± 703 | 704 ± 11.3 | 400 ± 47.6 | 326 ± 9.16 | 208 ± 5.16 | 144 ± 27.0 | 83.2 ± 32.8 | 12.6 ± 6.15 |
| K_p_ | / | 614 | 588 | 402 | 350 | 85.8 | 77.1 | 42.6 | 36.3 | 23.1 | 15.9 | 9.2 | 1 |

**Table S9** Metabolic stability of TET in RLM and HLM. *In vitro* metabolic stability of TET (1.0 μM) was examined in NADPH-supplemented liver microsomes from rat and human (0.5 mg/mL protein) for 60 min at 37°C (n = 3, Mean ± SD). Stability data governed by half-life (T_1/2_) was scaled to apparent intrinsic clearance (CL_int_) and hepatic plasma clearance (CL_h_) using the well-stirred model (detailed information see Eq. 6 and Eq. 7 in 2.10 part). Hepatic Extraction was the percentage of CL_h_ in physiological hepatic blood flow (55 mL/min/kg for rat, 21 mL/min/kg for human).

| Parameters | t_½_ （min） | CL_int_ （mL/min/mg protein） | CL_h_ （mL/min/kg） | Hepatic Extraction (%) |
| --- | --- | --- | --- | --- |
| RLM | 40.7 ± 3.1 | 61.5 ± 4.46 | 1.80 ± 0.13 | 3.26 ± 0.23 |
| HLM | 34.7 ± 4.0 | 46.7 ± 5.32 | 0.628 ± 0.07 | 3.04 ± 0.34 |
